# The brain and its time: intrinsic neural timescales are key for input processing

**DOI:** 10.1038/s42003-021-02483-6

**Published:** 2021-08-16

**Authors:** Mehrshad Golesorkhi, Javier Gomez-Pilar, Federico Zilio, Nareg Berberian, Annemarie Wolff, Mustapha C. E. Yagoub, Georg Northoff

**Affiliations:** 1 School of Electrical Engineering and Computer Science, University of Ottawa, Ottawa, Canada; 2 Mind, Brain Imaging and Neuroethics Research Unit, Institute of Mental Health, Royal Ottawa Mental Health Centre and University of Ottawa, Ottawa, Canada; 3 Biomedical Engineering Group, University of Valladolid, Valladolid, Spain; 4 Centro de Investigación Biomédica en Red en Bioingeniería, Biomateriales y Nanomedicina, (CIBER-BBN), Madrid, Spain; 5 Department of Philosophy, Sociology, Education and Applied Psychology, University of Padova, Padua, Italy; 6 Centre for Cognition and Brain Disorders, Hangzhou Normal University, Hangzhou, China; 7 Mental Health Centre, Zhejiang University School of Medicine, Hangzhou, Zhejiang China

**Keywords:** Computational neuroscience, Cognitive neuroscience

## Abstract

We process and integrate multiple timescales into one meaningful whole. Recent evidence suggests that the brain displays a complex multiscale temporal organization. Different regions exhibit different timescales as described by the concept of intrinsic neural timescales (INT); however, their function and neural mechanisms remains unclear. We review recent literature on INT and propose that they are key for input processing. Specifically, they are shared across different species, i.e., input sharing. This suggests a role of INT in encoding inputs through matching the inputs’ stochastics with the ongoing temporal statistics of the brain’s neural activity, i.e., input encoding. Following simulation and empirical data, we point out input integration versus segregation and input sampling as key temporal mechanisms of input processing. This deeply grounds the brain within its environmental and evolutionary context. It carries major implications in understanding mental features and psychiatric disorders, as well as going beyond the brain in integrating timescales into artificial intelligence.

## Abstract (web summary)

Golesorkhi et al. discuss recent literature on intrinsic neural timescales, their potential role in input processing including computational mechanism, and how they relate to mental features, psychiatric disorders and artificial intelligence.

## Introduction

Our environment bombards the brain with a variety of regular and irregular inputs on different timescales. Consider one of the temporally most complex inputs, music. We can simultaneously perceive the music’s different timescales and, even better, are able to integrate them into one meaningful whole like a melody. Moreover, the melody can be distinguished from the ongoing accompaniment in the background.

How can our brain process and integrate such multiscale inputs? Recent evidence suggests that the brain itself exhibits intrinsic neural timescales (INT)^1–11^. As measured by the autocorrelation window (ACW) in resting state, lower-order unimodal sensory regions—the primary visual cortex, for example—shows short timescales compared to higher-order transmodal regions like the default-mode network (DMN)^2–10,12–23^. However, the specific function or role of the INT in the brain and its neural processing still remains unclear.

**Fig. 1:**
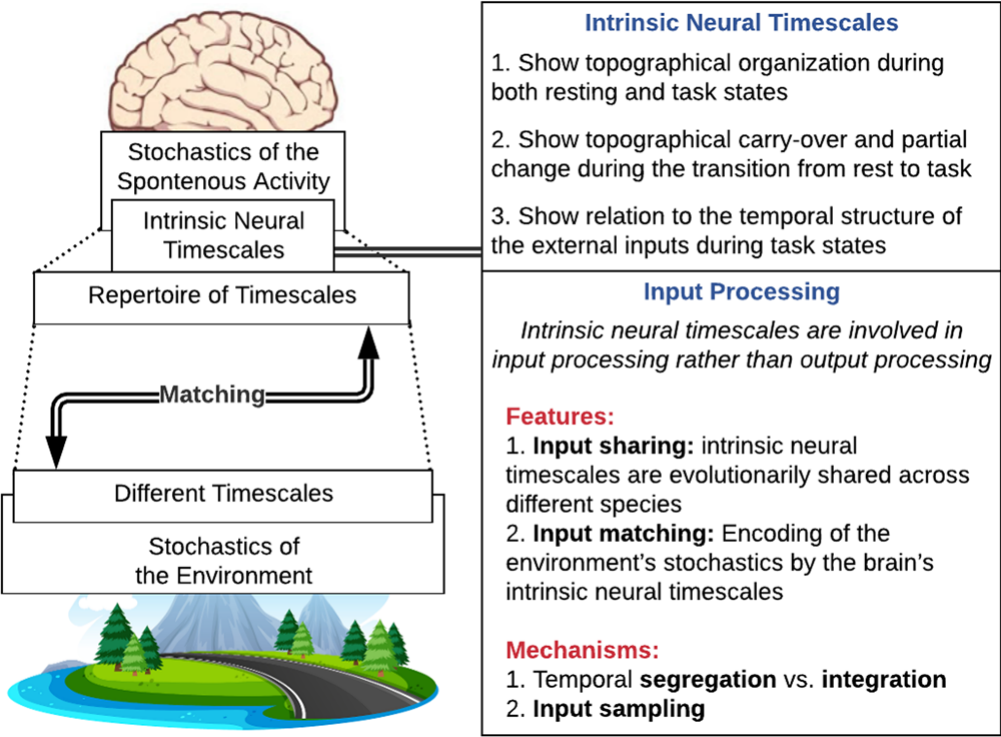
The proposed function of the intrinsic neural timescales in input processing. The key assumption is that the intrinsic neural timescales process the input by matching the stochastics of neural activity with the stochastics of the environment. The nature clip art credit: Nature Vectors by Vecteez (https://www.vecteezy.com/free-vector/nature).

Reviewing various findings on INT in both human and non-human species, we propose that they play a key role in processing and structuring inputs in different timescales (Fig. 1 for a general framework). Rather than focusing on specific kinds of inputs (like visual, somatosensory, or auditory; see Box 1), we here aim to describe basic dynamic principles of the temporal nature of input processing that are shared across different inputs. Specifically, the brain utilizes its own INT to process and actively shape the extrinsic timescales of the multiscale inputs it receives from both environment and body. This allows the brain to encode the stochastic structure of its environmental inputs according to its own stochastic structure. Its own stochastic structure is determined by its INT and its unimodal–transmodal hierarchy. That, as we will detail, is mediated by specific computational mechanisms like temporal integration/segregation as well as input sampling with consecutive shifting towards slower frequency modes within the processing hierarchy.

We first review empirical evidence on INT during both resting and task states. That is followed by a second part where we link INT to distinct facets of input processing, like encoding of stochastics and their sharing by different species. In the third part, borrowing from Physics and Mathematics, we explore the computational mechanisms driving input processing by the INT; this includes temporal integration and segregation of the input, as well as a sampling mechanism that shifts subsequent stages of input processing towards slower frequency modes. We conclude that the role or function of INT consists in processing and structuring multiscale inputs from the environment which, to some degree, is evolutionarily shared across human and non-human species. Given its key role in the brain’s input processing, INT carries major implications for psychiatric disorders (Box 2) and artificial intelligence (Box 3).

Box 1 Inputs: deeper dynamic levels of input processing The notion of input is a concept that can include different meanings. One first and foremost associates sensory functions with the notion of input. For instance, one can distinguish somatosensory, visual, and auditory inputs. Recent studies^25,116^ demonstrate that these sensory input systems are related, with different input streams in the brain in terms of both their functional connectivity^116^ and INT^25^. Most interestingly, these input streams share continuous progression from unimodal (like primary sensory cortex) to more transmodal regions like the dorsolateral prefrontal cortex. The transmodal regions are, in part, shared by the different sensory input systems.

Hence, despite their distinctions in terms of their modality-specific sensory input, these input systems may nevertheless converge in the higher-order transmodal regions. Such convergence of the sensory input streams with the unimodal–transmodal hierarchy of INT suggests that some dynamic, i.e., temporal features and mechanisms of input processing, as the ones discussed above, may be shared among the different sensory modalities.

In addition to sensory inputs from the external environment, the brain also receives inputs from the body, that is, its interoceptive inputs (and also its proprioceptive inputs). Taylor et al.^116^ demonstrate that the brain’s interoceptive input streams (with insula as the key region) again follow the hierarchical progression from unimodal to transmodal regions. Remarkably, even the interoceptive input system converged with the exteroceptive sensory input streams in the higher transmodal regions. That suggests some commonality among intero- exteroceptive input processing.

Complementing the continuous “bombardment” by the environment’s exteroceptive inputs and the body’s interoceptive inputs, the brain itself shows activity changes in its spontaneous activity. These changes may be weak or, alternatively, stronger, inducing the degree of activity changes usually elicited by exteroceptive stimuli. This may, for instance, apply to spontaneous activity changes in the auditory cortex which then may be perceived as external voices while being hallucinatory in their nature^77,117,118^. These spontaneous activity changes may even exist in the “healthy” brain (as healthy persons can show auditory hallucinations) and may therefore be designated as “neuronal input”^77^. These spontaneous changes may be predominantly associated with internally oriented cognition like mind-wandering^119,120^, self-referential processing^121,122^, and mental time travel/episodic simulation^67,112^. Importantly, these spontaneous activity changes, the neuronal inputs, occur in all regions of the brain and, interestingly, are significantly stronger in the higher-order transmodal regions when compared to the unimodal regions^106,123–125^. Hence, we can observe again that even the brain’s own neuronal input seems to follow the progression from the unimodal to the transmodal hierarchy.

Together, these observations suggest that beyond the distinction between specific sensory inputs, as well as the different sources of inputs (environment, body, brain), there is some deeper level to the notion of input. Unlike the more superficial level with the distinction of different inputs and sources, there seems to be a deeper level that is shared among the different inputs. This deeper level of the input seems to be strongly shaped by the brain’s INT in strongly temporal and thus dynamic way. Our description of the different features and mechanisms of input processing in this paper targets this deeper temporal, highly dynamic level of input processing.

Box 2 From intrinsic neural timescales to psychiatric disorders Are INT relevant for behavior and cognition? Evidence for that comes from their changes in psychiatric disorders. A recent resting state fMRI study^14^ applied the ACW in subjects with autism. They observed significantly shorter ACW in primary sensory regions (visual, sensorimotor, auditory) in adult autism spectrum disorder (ASD) compared to healthy subjects; these changes correlated negatively with the severity of autism. In contrast, ACW in right caudate was significantly longer in ASD which also correlated with the degree of repetitive restrictive behavior in ASD subjects.

They then investigated fMRI resting state ACW in an adolescent children ASD dataset where they obtained similar ACW changes in the same regions including analogous correlation results. This means that there is a developmental component to the intrinsic timescales. That was complemented by investigating the neuro-anatomical basis through calculating the local gray matter volume. Significant positive correlation of local gray matter volume with the duration of ACW in the same region was observed which also hold for the regions altered in ASD. Finally, they calculated mediation analysis showing that the gray matter volume in the above-mentioned regions was mediated in their impact on autistic symptoms by the duration of the ACW^14^.

The relevance of intrinsic timescales in autism is further supported by another fMRI resting state study in ASD^126^. Operating in the frequency domain (rather than time domain), they calculated the power-law exponent (PLE) (and spectral entropy). They observed that ASD showed increased PLE with stronger power in slow frequencies in specifically regions of the salience network (insula, supragenual anterior cingulate cortex, and thalamus) (see also ref. ^127^ showing that the salience network exhibits the highest variability and flexibility among the different networks). Moreover, they demonstrated that such increased PLE in salience network was not observed in schizophrenia. Finally, these findings were specific for PLE as distinguished from others like regional homogeneity and neural variability that did not show any changes in these regions in ASD^61,128,129^.

Using EEG, one recent study in schizophrenia including mostly post-acute first-episode subjects showed abnormally long ACW (and high PLE) in several electrodes during a task state involving self-specificity (i.e., enfacement task)^130^. They also demonstrated that the degree of change in ACW from rest to task was significantly lower in schizophrenia subjects, that is, unlike in healthy subjects, they barely shortened their ACW during the task. Most remarkably, analogous ACW prolongation during task and its reduced rest–task difference was not observed during a non-self-task, i.e., auditory oddball—this suggests a close relationship of ACW with self-specificity.

Moreover, applying a moderation model, they showed that the degree of ACW mediated the relationship between self-disturbance and negative symptoms in schizophrenia participants. As in the autism study by Watanabe and colleagues^14^, these data support the assumption that changes in INT may be related to psychopathological symptoms and, more generally, be relevant for behavior and cognition. This is further supported in a recent study on psychosis in schizophrenia^25^.

Using fMRI, they show globally reduced (i.e., shortened) INT in psychosis/schizophrenia compared to healthy controls. Moreover, they observed specific changes within the neural hierarchy of auditory and somatosensory input streams: the increase in INT at lower levels of the neural hierarchies may reflect hallucinations (comparing psychotic subjects with severe vs. mild hallucinations). While the increased timescales at higher levels of neural hierarchies may reflect delusions (comparing psychotic subjects with severe vs. mild delusions). Together, the findings by^25,130^ support the importance of INT in mediating specific psychopathological symptoms of psychosis.

**Figure:**
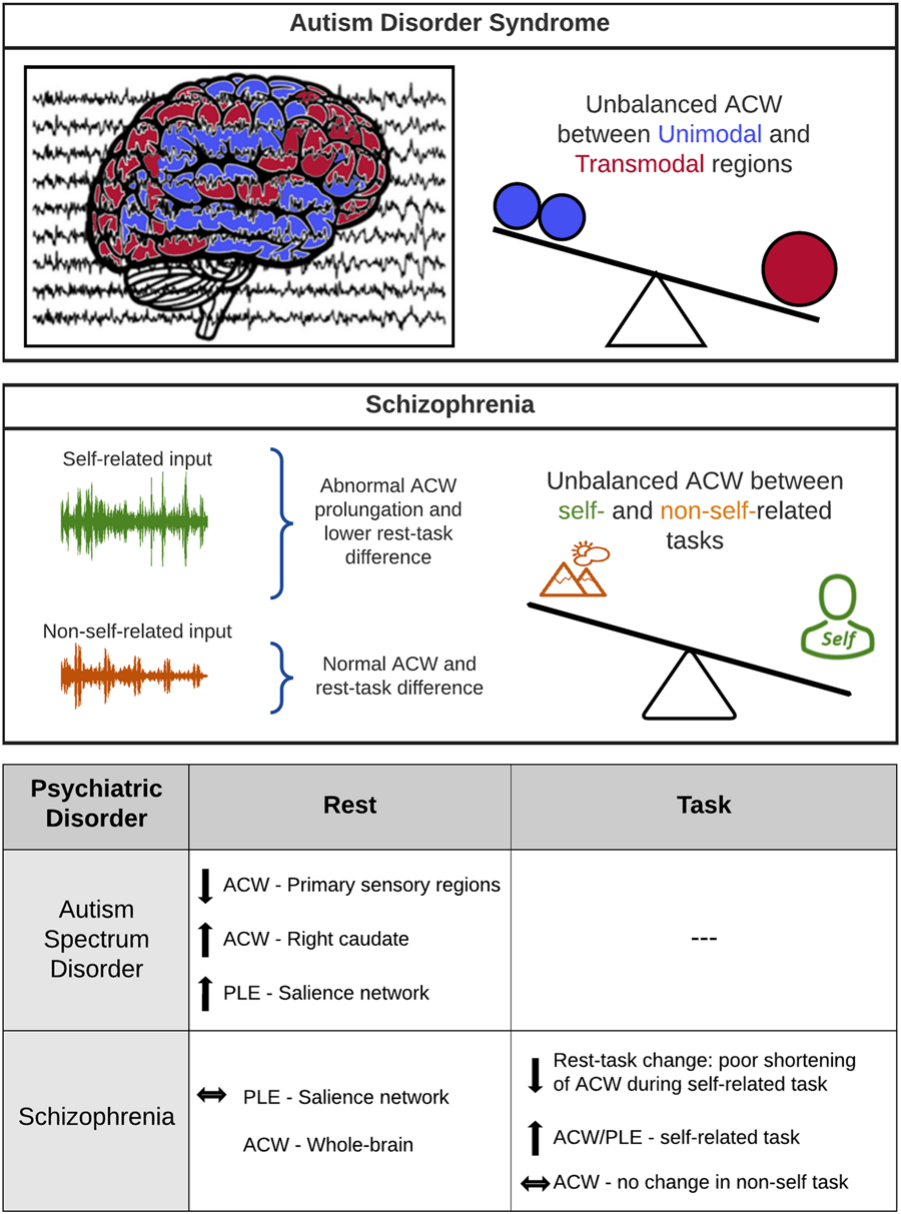
Unbalanced ACW in unimodal vs. transmodal regions and self-related vs. non-self-related tasks. **Figure of Box 2:** The different lengths of ACW observed in subjects with autism and schizophrenia compared to healthy subjects suggest that changes in the intrinsic neural timescales may be associated with particular psychopathological symptoms of these disorders.

Box 3 From intrinsic neural timescales to artificial agents We saw that the environmental hierarchies of different events may be recapitulated and thus modeled by the brain itself within its own intrinsic hierarchical organization, i.e., its temporo-spatial hierarchy. There is no need for the living to “represent” a model of the environment in their head: “An agent does not have a model of its world—it is a model. In other words, the form, structure, and states of our embodied brains do not contain a model of the sensorium—they are that model”.^131^. Based on the temporal hierarchy of its INT, the human agent (and related non-human species) is a temporo-spatial model of its environment displaying a more or less analogous temporal structure albeit in a miniature scale-free way.

The brain can be conceived as a free energy-driven temporo-spatial model of the temporo-spatial hierarchies of its environmental context. That results in temporal and spatial nestedness of the brain within its respective environmental context. Despite different temporal (and spatial) scales across body, brain, and environments, they are nevertheless connected through scale-free self-similarity in their shape or form. Just like the smaller Russian doll is contained within the next larger one (same shape, different size), the brain and its temporo-spatial organization nest in a scale-free self-similar way within the much larger environment^132^. Given such temporo-spatial self-similarity between brain and environment, we may better focus on “what our head’s inside of” rather than searching for “what inside our heads”^86^.

This carries major implications for modeling artificial agents as it entails the need to include an intrinsic temporal and spatial hierarchy within the inner design and architecture of the agent itself. Future AI models may want to implement such intrinsic spatial and temporal organization in their artificial agents, including the different timescales and the core–periphery organization (see ref. ^133^ for first steps in this direction in artificial agents using what they describe as “multiple time scale recurrent neural network”, refs. ^134,135^ who emphasize the need for temporal hierarchies in artificial agents for their adaptation to the environment).

Spatiotemporal hierarchies would extend the current—often module-based—models of artificial agents^136^ by combining top-down (providing the agent’s inner input) and bottom-up (providing the agent’s outer input) layers—that Tani uses in his compelling model of an artificial agent^135,137,138^. Most importantly, by conjoining the temporo-spatial architecture with the free energy principle, the artificial agent’s intrinsic temporo-spatial hierarchy may be a small-scale but self-similar model of its own environmental context. To achieve that, the agent’s temporo-spatial hierarchy needs to be highly dynamic and continuously changing, so as to adapt to the changing environmental dynamics. More specifically, this means that the causal (or temporo-spatial) architecture of the environment must be recapitulated or installed in the agent’s temporo-spatial dynamics in such a way as to allow the agent to minimize its variational free energy with its respective environmental context.

**Figure:**
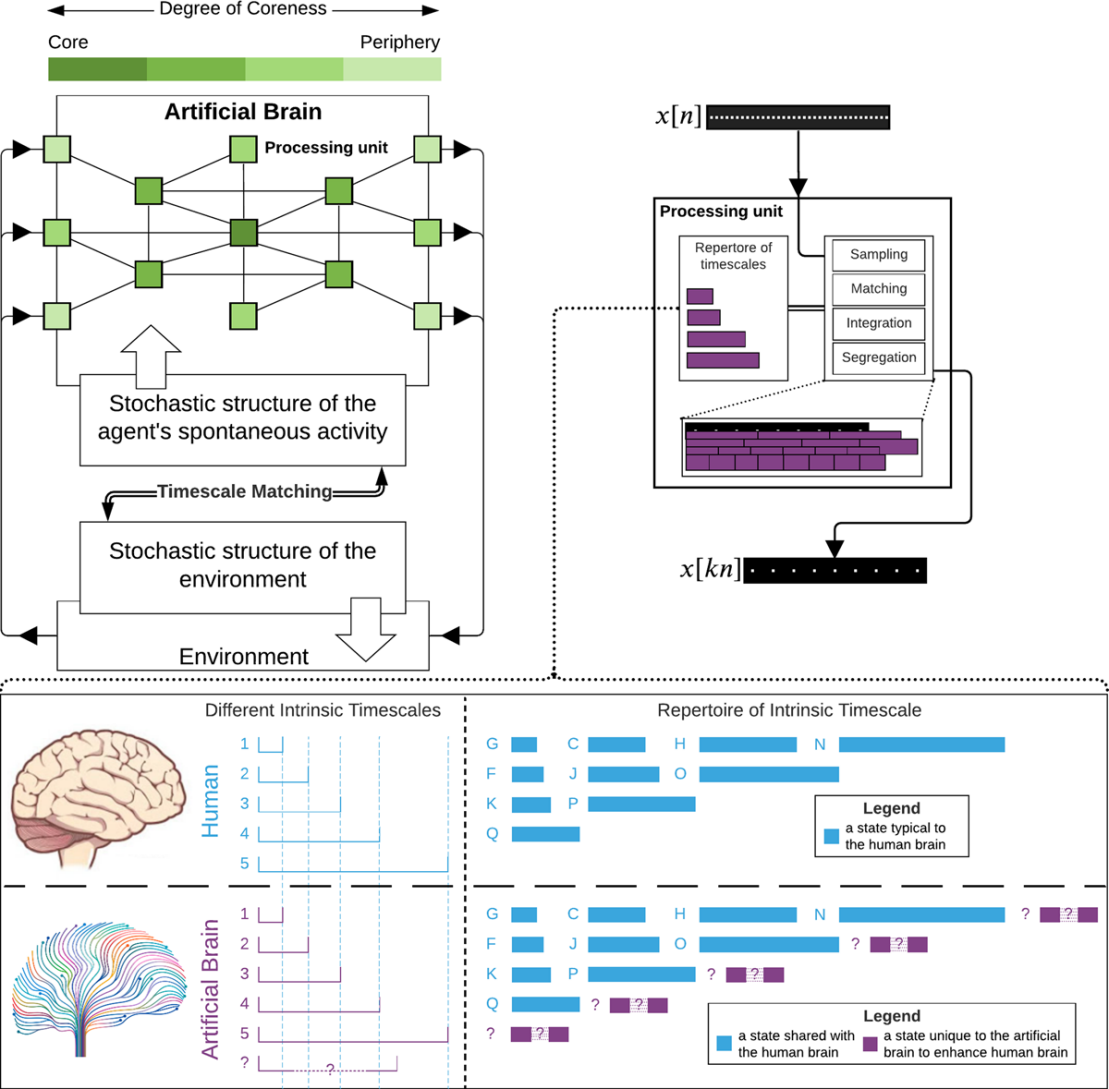
A suggested schematic of agent-environment interaction. **Figure of Box 3:** The agent and the environment are in a closed loop with the environment affecting the agent and vice versa. The stochastic of the agent’s spontaneous activity acts as its interface and interacts with the environment’s interface, i.e., its stochastic structure, through timescale matching. The agent’s processing units are organized in a core–periphery structure. In each processing unit, the input is sampled, matched, integrated, or segregated based on the intrinsic properties of the unit.

## Part I: intrinsic neural timescales in rest and task states

### Calculation of intrinsic neural timescales

INT is commonly investigated in cellular^4,24^ and systemic^1,12–14,22,25,26^ granularity levels. On a systemic level, Hasson and colleagues^1,12,26,27^ operationalize INT using functional connectivity during task state. They define temporal receptive windows as “the length of time before a response during which sensory information may affect that response”^1^ and it roughly correspond to what are described as temporal receptive fields on the cellular level^28^.

Recent studies operationalize the INT using the autocorrelation function of the signal during both resting and task state. Autocorrelation function is the correlation of a signal with shifted (time-lagged) versions of itself^4^. Since autocorrelation function yields a series of numbers, different studies report slightly different properties of it. Murray and colleagues^4^ obtain INT by fitting an exponential curve to the autocorrelation; however, ACW is most commonly reported in the INT’s literature.

**Fig. 2:**
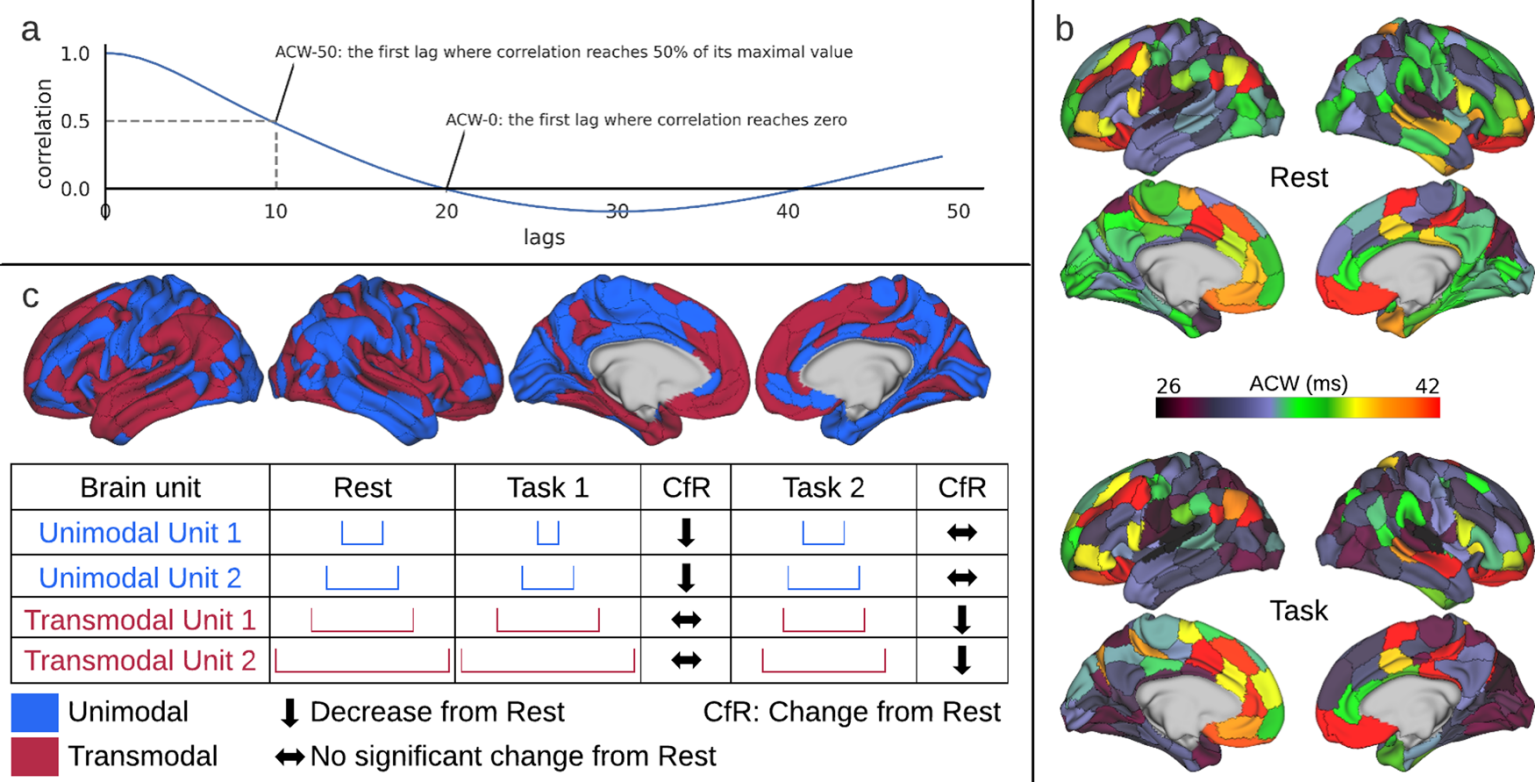
Autocorrelation window (ACW) in resting and task states. **a** ACW is defined as the first lag in which the correlation of the signal with itself drops below 50% of the maximum correlation. It is measured from the autocorrelation function. **b** The ACW, as recorded in MEG^22^, shows topographical differences between regions during resting and task states. The colormap is in milliseconds and represents the length of ACW. **c** The brain map represents the uni-transmodal organization of the brain regions. The table schematically shows how ACW changes from resting to task states and also from unimodal to transmodal units in two arbitrary tasks and four sample units. The table is only for illustration purposes. Unimodal and transmodal units refers to either unimodal or transmodal regions in the brain. The numbers 1 and 2 indicate the hierarchical position (1 = lower; 2 = higher) of the respective region/unit. The blue (unimodal) and red (transmodal) intervals represent the width of their respective intrinsic neural timescales (INT) during rest and two different tasks (task 1 and 2). CfR indicates the change from rest to task with either decreasing (downward arrow), increasing (upward arrow), or maintained (horizontal double-sided arrow) width of the regions’ INT during task relative to rest. Though schematically, the differences in the width and rest–task change of the INT during the two tasks shall indicate the flexible and adaptive nature of the timescales as it is supported on both regional^22^ and cellular^24,36,37^ levels (see in rest–task overlap and rest–task modulation sections).

In their fMRI studies, Watanabe and colleagues^14^ define ACW as the area under the curve of the autocorrelation function from zero to the time lag that the correlation reaches zero (see also refs. ^13,25^). However, EEG/MEG studies define ACW as the time lag the correlation reaches half of its maximum value (Fig. 2a) or when it reaches 1⁄*e* (Golesorkhi et al.^22^ defines a new variant called ACW-0).

### Resting state I: temporal hierarchy of unimodal and transmodal regions

Murray and colleagues^4^ investigated single-cell recording data in non-human primates and calculated their autocorrelation function in pre-stimulus intervals. From that, they measured the duration of the temporal window at a correlation decay of 50%, i.e., ACW. They observed a shorter ACW in lower-order unimodal sensory regions while higher-order transmodal regions, such as the prefrontal cortex, exhibited a longer ACW^4^. Subsequent computational modeling studies employed large-scale non-human primate-based, human-based structural connectivity networks^5,29^, or a standard model of synchronization, i.e., Kuramoto model^2^ (0.01–0.1 Hz). They also demonstrated longer INT (as measured by the ACW) in prefrontal regions which remained shorter in sensory and motor cortex (see also ref. ^17^, see also other models of neural dynamics in refs. ^30,31^).

The computational findings are supported by observations of a corresponding hierarchy of timescales in real human data using fMRI^13,18^. Operating in the infraslow frequency range (0.01–0.1 Hz), resting state fMRI studies applied the autocorrelation function to the BOLD signal and, following Murray and colleagues^4^, determined the ACW at 50% of correlation decay^13,14,18^. Employing small^14,15^ or large-scale^13,18,29^ fMRI datasets, all studies observed shorter ACW in unimodal regions, including sensory and motor regions/networks on the cortical level. In contrast, transmodal regions, including higher-order networks such as the central-executive networks (CEN), dorsal attention networks (DAN), and default-mode network (DMN), generally show a longer ACW.

In addition to a temporal hierarchy on the cortical level, Raut and colleagues^13^ also measured the ACW in subcortical regions like the thalamus, cerebellum, striatum, and hippocampus. Interestingly, they again observed that gradients of the ACW within each of these subcortical regions, especially in the thalamus and striatum, appear to mirror the temporal hierarchy on the cortical level. Together, these data strongly suggest that both cortical and subcortical regions display an intrinsic hierarchical organization with unimodal sensory and motor regions showing shorter timescales while transmodal higher-order association regions exhibit longer timescales.

These findings were all obtained in human fMRI that measures BOLD activity in the infraslow frequency range (0.01–0.1 Hz). That raises the question as to whether the distinction of shorter unimodal and longer transmodal INTs are also present in faster frequency ranges between 1 and 70 Hz as can be typically measured with EEG/MEG. Indeed, two recent human resting state MEG studies^22,29^ demonstrate a longer ACW in higher-order transmodal regions/networks like the CEN and DMN, whereas it was significantly shorter in unimodal sensory regions. Hence, these findings suggest that INT follows a similar topographical distribution in faster frequencies (1–70 Hz) as those in slower frequency ranges (0.01–0.1 Hz). Such ubiquitous occurrence suggests a most basic or fundamental, though unclear, role or function of INT in the brain.

### Resting state II: intrinsic neural timescales and functional connectivity

How are the intra-regional INTs related to inter-regional connections? The INT is constituted by both intra-regional cellular features^5,16,32,33^ and inter-regional connectivity^4,5,13,20^. Intra-regional cellular features concern the excitation–inhibition balance with its local recurrent wiring^34^ as in supragranular feedforward and infra-granular feedback connections^5,13,32,35^ (see also ref. ^20^ for demonstrating the relevance of population codes). Cavanagh et al.^36^ demonstrate that even within regions there is considerable variability of the INT at the single neuron level. Specifically, the temporal receptive field of a single neuron can change over time and adapt to, for instance, task demands as during working memory (see also ref. ^24^) and/or decision making^36–38^. Moreover, Spitmaan et al.^37^ observe less dependence upon the task context—this further underlines their adaptative nature. Moreover, as the authors put it, the timescales of different neurons during task-related activity suggest a certain independence, i.e., flexibility.

In addition to the intra-regional cellular features, INT is also strongly shaped by inter-regional connectivity. Chaudhuri and colleagues^16^ demonstrated that purely local connectivity itself is insufficient to yield the diversity of timescales across the cortex. Moreover, in their non-human primate-based computational model^5^, they remove all long-range projections which significantly restricts the range of different timescales and abolishes the intrinsic temporal hierarchy. The relationship of intra-regional INT and inter-regional functional connectivity holds again across different species as it can be observed in both non-human primates^9^ and humans^13,18,29,39–42^.

How exactly is inter-regional functional connectivity related to the INT? Two recent studies in human fMRI show that the duration of INT in different regions, as measured by the resting state ACW, is positively correlated with the degree of said region’s change in functional connectivity during task: the longer the region’s resting state ACW, the stronger its task-related change in its functional connectivity to other regions^18,43^. That is further supported by Raut and colleagues^13^ who demonstrated that the individual variability in ACW across different regions is directly related to the individual variation of the functional connectivity pattern of the same regions (see also^42,44–48^).

Together, these findings suggest a close relationship of INT to the brain’s inter-regional connectivity pattern—intra-regional temporal features are, in part, constituted by long-range inter-regional connections. Such an intimate link between intra-regional timescales and inter-regional connectivity means that the different timescales can interact and integrate with each other. This may enlarge the number of available timescales, i.e., the repertoire of timescales, as we will illustrate later.

### Rest–task overlap: from intrinsic neural timescales to temporal receptive windows

Is the resting state’s INT related to task states? A positive answer to this question would support their involvement in input processing. The relevance of INT for input processing is strongly suggested by the excellent studies of Hasson and colleagues^15,26,27,49–51^ (ref. ^1^ for review). They demonstrate that shorter temporal segments of external stimuli (like single words of stories, or short episodes in movies) are processed preferentially in lower-order unimodal sensory regions. Longer intervals (like whole paragraphs in stories, or longer episodes in movies) are related to activity changes in higher-order transmodal regions. Given that external inputs are processed and structured in temporal terms, i.e., according to different durations, Hasson and colleagues^1^ speak of temporal receptive windows which roughly correspond to what are described as temporal receptive fields on the cellular level^28^.

Does the spatial, or topographical, pattern of the INT overlap between rest and task states, i.e., rest–task overlap? While such rest–task overlap has been well demonstrated for functional connectivity^18,52–55^, it remains an open issue in the case of INT. The various task studies on the brain’s temporal receptive windows show a spatial pattern that is well in accordance with the hierarchical organization of INT in the resting state. In the same way, the ACW is longest in the DMN during rest. Task states also show that the DMN processes the longest sequence of inputs and information^26,27^, while the shorter resting state ACW in unimodal sensory regions seems to find its equivalent in the short sequences of inputs processed in these regions^1^. Hence, comparison of rest ACW and task temporal receptive windows shows analogous hierarchical topographical organization. This suggests a close relationship between rest and task, i.e., rest–task modulation or interaction^56–60^ (see below for the discussion of task-specific changes in INT).

If there is such a rest–task overlap, one would assume that the hierarchical organization of resting state ACW is carried over to, and thus present in, the temporal receptive windows during task states. Evidence for such rest–task overlap comes from both computational modeling and brain imaging. Gollo and colleagues^2^ conducted a modeling study based on the synchronization model of Kuramoto with simulations of transcranial magnetic stimulation: they show that regions with longer ACW, as located in the transmodal core, display lower and more sluggish activity changes in response to external stimuli than sensory regions; the latter exhibit a shorter ACW at the more unimodal periphery, accompanied by higher amplitude and a faster response to external stimuli (see also refs. ^17,35^). Analogous results were observed in the modeling study by Chaudhuri and colleagues^5^ who applied electrical stimulation to V1 in the visual cortex to his non-human primate-based network model (see also ref. ^29^). One interesting finding here is that regions weakly connected to the input regions exhibit longer INT during stimulation. This again demonstrates that tasks exert effects beyond those at the stimulated regions themselves. These computational data on rest–task overlap of INT are supported by human brain imaging data. A recent human fMRI study by Ito and colleagues^18^ investigated the ACW in resting state and the amplitude during different task states. They demonstrated a negative correlation of resting state ACW duration (in different regions) with the magnitude of task-related activity, i.e., amplitude, in the same regions. Therefore, the longer the region’s resting state ACW, i.e., transmodal regions, the lower its task-related amplitude. While regions with shorter ACW, i.e., unimodal regions, exhibit higher amplitude during different tasks. These results support the idea that the resting state’s INT strongly shapes task-related activity and associated input processing^2,60,61^. The mechanisms of this, however, remain unclear.

### Rest–task modulation: intrinsic neural timescales shape task states and behavior/cognition

The rest–task overlap strongly suggests that the resting state’s INT may also shape or modulate the temporal features of task states including associated cognition—this amounts to what we describe as rest–task interaction or modulation (see also refs. ^56,58,62^). This has recently been addressed by Golesorkhi and colleagues^22^ (see also ref. ^15^ for initial steps). Applying MEG, they investigated the ACW-50 and ACW-0 (see above) not only during rest but also during three different task states (motor, story-math, working memory). They showed that the resting state’s ACW and its hierarchical core–periphery organization strongly predict their task states: the resting state’s core–periphery organization of ACW was essentially preserved during all three task states as topographical rest–task correlation yielded high values (0.8–0.9)^22^. These results suggest that the resting state’s hierarchical organization of its INT is essentially carried over, and preserved, during task states, irrespective of the task.

Golesorkhi and colleagues^22^ also observed some task-specific changes (Fig. 2) when calculating the rest–task difference (which subtracts and cancels out the shared, i.e., correlating temporal hierarchical organization). Specifically, higher-order network regions showed a strong ACW, shortening during the story-math task (which was presented in 30 s intervals). Only minimal changes were seen in motor and working memory tasks. A reverse pattern was observed in lower-order network regions; the ACW was shortened considerably during working memory but minimally in story-math and motor tasks. These data suggest that, once one subtracts the hierarchical temporal organization present in both rest and task, task-specific changes can be observed. Furthermore, the ACW itself and, more generally, the INT, can be modulated during task states—they are dynamic and adaptive rather than static and non-adaptive. Though more studies are needed, task-related modulation seems to mainly concern the shortening of the ACW relative to rest. The adaptative nature of INT is also documented by them during the delay period of a working memory task (relative to pre-stimulus baseline) in human ECoG^24^.

In addition to task states, INT also shape behavior and cognition. Studies in non-human primates demonstrated that a longer duration of the resting state’s INT (as obtained during baseline intervals sandwiched between tasks) is associated with better behavioral performance in a variety of different tasks. These include a longer duration of delays in a delay discounting task^4^, stronger spatial response coding in the delay period during a non-match-to-goal task^63^, and modulating working memory performance during later periods, i.e., delay^9^. On the human side, recent fMRI and/or EEG studies demonstrate that the resting state’s ACW is directly related to higher-order cognition like the level of consciousness^64,65^, the sleep stage^21^, the sense of self^66–69^, and psychiatric disorders (see Box 2 for details). Tentatively, these data show that INT strongly shapes behavior, including perception and higher-order cognition like consciousness and self. Since task states, as well as perception and cognition, are dependent upon various kinds of inputs, together these data are compatible with the assumption that INT is key for input processing and structuring.

## Part II: input processing through intrinsic neural timescales

Key findings of the INT are: (I) their topographical organization during both resting and task states along uni- and transmodal regions/networks; (II) their topographical carry-over and partial change during the transition from rest to task^22^; and (III) their relation to the temporal structure of external inputs during task states^1,26,49,50,70,71^. Together, these findings suggest their involvement specifically in the brain’s input processing.

We presuppose here a wide notion of input including stimuli from both one’s own body, i.e., interoceptive, and external environment, i.e., exteroceptive (Box 1). Our focus is primarily on the dynamic principles and mechanisms underlying input processing in general rather than describing the specifics of various inputs like auditory, visual, or somatosensory (see Box 1). Considering INT, in the following we first address the importance of input processing as distinguished from output processing and then discuss two important facets: (I) cross-species input sharing (II) and stochastic matching of the extrinsic environmental inputs with the brain’s intrinsic stochastic structure.

### Input vs. output: capacity or predisposition for input processing

Is INT engaged in either input or output processing? This was recently addressed by Zilio and colleagues^21^, who investigated the ACW in resting state EEG in subjects with physiologic, pharmacologic, and pathological alterations of consciousness. Under such conditions, input processing is known to be altered in distinct ways, i.e., progressive decrease (NREM sleep stages N1, N2, N3), isolation from external inputs but preserved capacity for processing of internal inputs (from the own body and brain) (REM sleep and ketamine), and extreme deficiency or complete absence of both external and internal inputs (unresponsive wakefulness syndrome, sevoflurane). Additionally, they included subjects with complete loss of motor function, e.g., output processing, whereas input processing and consciousness are preserved (locked-in syndrome and amyotrophic lateral sclerosis).

**Fig. 3:**
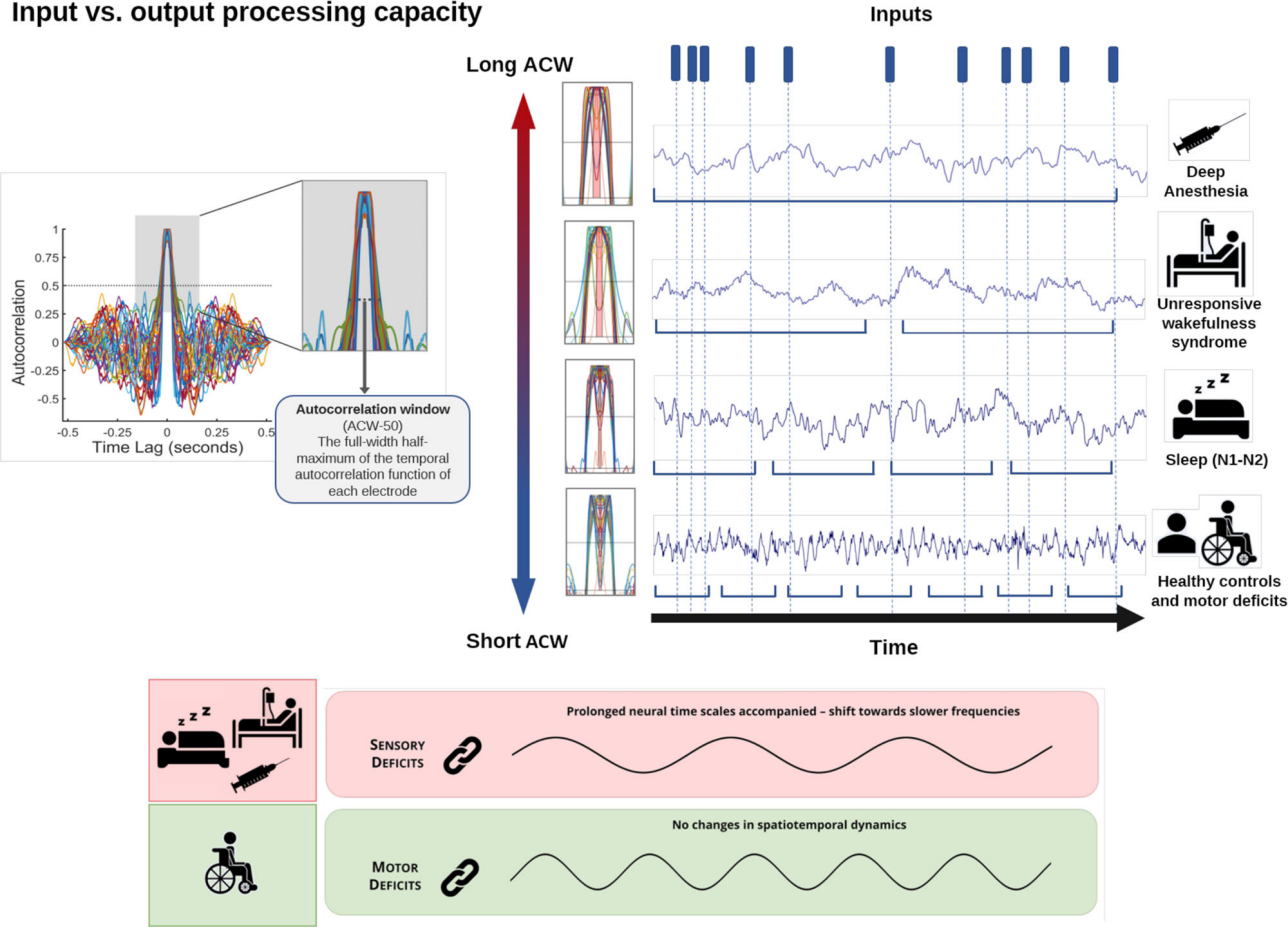
Input vs. output processing. On a whole-brain level, healthy awake subjects and subjects with motor deficits but preserved input processing (amyotrophic lateral sclerosis, locked-in syndrome) present short ACW, i.e., normal neural timescales accompanied by a balance of slow and fast frequencies, which is associated with a normal capacity of encoding inputs, while other physiological, pharmacological, and pathological conditions, e.g., sleep (N1-N2), unresponsive wakefulness syndrome and deep anesthesia (e.g., sevoflurane) show a progressive stretching of the ACW, i.e., prolonged neural timescales accompanied by a shift towards slower frequencies, which consequently lead to the abnormal prolongation of the input processing temporal window (the EEG signals and the ACW representations are taken from the datasets investigated in Zilio et al.^21^).

The results (Fig. 3) show abnormally long ACWs in the unresponsive wakefulness syndrome, through abnormal strengthening of slow frequency power (and concurrent weakening of fast frequency power). Also, both the physiologic and pharmacologic alterations of consciousness showed abnormal prolongation of the ACW in line with the progressive decrement of the capacity of input processing in the different behavioral states. The motor conditions, in contrast, exhibited a “normal” ACW with a preserved balance of slow and fast frequencies in the power spectrum. Together, these findings support the involvement of INT specifically in input processing. In contrast, INT does not appear to be significantly associated with output processing in subjects with motor deficits but preserved input processing. If the ACW was significantly involved in both input and output processing, it should have globally changed in both types of conditions, altered states of consciousness and altered motor conditions (although the ACW is significantly shorter in the parieto-occipital regions of amyotrophic lateral sclerosis patients than in healthy subjects). We need to be cautious, however. One can neither fully exclude output disturbances in the altered states of consciousness nor changes in input processing in the motor conditions (locked-in syndrome, amyotrophic lateral sclerosis). Hence, more direct support for the role of the ACW in input processing is required.

Of note, however, is that Zilio and colleagues^21^ investigated only resting state activity. Therefore, the ACW can only be indirectly related to input processing; an investigation of task states with actual inputs are needed to provide a direct relation to input processing. Given that the disorders and the alterations of consciousness are known to exhibit deficient input processing^72–76^, their findings suggest that the resting state’s INT exerts the capacity for input processing, i.e., a neural predisposition^77–80^. Even when not exposed to actual multiscale inputs from the external environment, the resting state still exhibits its own INT, which index its capacity for processing the former. This is, for instance, the case in sleep where we can still be awoken at any time by sufficiently strong external inputs—the brain’s capacity or predisposition of input processing is preserved^76^. In contrast, this remains impossible in total anesthesia and coma where even the strongest external inputs will not wake the individual—the brain’s capacity or predisposition of input processing is lost.

### Input sharing: cross-species evolutionary preservation of intrinsic neural timescales

We so far demonstrated the significance of INT in processing inputs from the external environment. Assuming that different species somewhat share one and the same external environment, then one would suppose that they should, to some degree, overlap or share, at least in part, their INT. There is indeed evidence for such “input sharing” across species as it is manifested in the cross-species evolutionary preservation of INT.

The data suggests that the regional differentiation of the INT along the transmodal–unimodal gradient holds in both non-human primates^4,5,20^ and humans^13,14,18,22^. This can be extended to other species as it is supported by cross-species studies on both the cellular^81,82^ and more regional-systemic^83^ levels. Shinomoto and colleagues^81,82^ show, in a first step, regional differentiation in the cellular firing pattern of different regions in the non-human primate brain: spiking patterns are regular in motor areas, random in visual areas, and burst-like in the prefrontal cortex. In a second step, they demonstrate that such temporal fingerprinting in the regions’ temporal structure of their firing pattern holds across different species including non-human primates, cats, rats, and mice; the differences in firing patterns between different regions within one species are larger than the firing pattern differences within the same region across different species^82,84^. Together, these data demonstrate that temporal features of neural firing patterns on the cellular level of specific regions are preserved across different species.

Analogous observations of cross-species evolutionary preservation have been made on the more regional-systemic level of oscillations. Buzsáki and colleagues^83^ demonstrate that various oscillatory rhythms such as alpha, spindles, and ripples are present in more or less the same frequency range in different species including humans, non-human primates, dogs, bat, gerbil, guinea pig, rabbit, mouse, and hamster (see also ref. ^84^). Importantly, Buzsáki and colleagues^83^ observe that such preservation of the same frequencies across different species holds independent of brain size: even if the brain size changes and becomes larger throughout evolution, the frequency range of the rhythmic pattern remains the same in different mammals. They conclude that temporal organization of the brain is a key priority in evolution: “In summary, the preservation of temporal constants that govern brain operations across several orders of magnitude of timescales suggests that the brain’s architectural aspects—scaling of the ratios of neuron types, modular growth, system size, inter-system connectivity, synaptic path lengths, and axon caliber—are subordinated to a temporal organizational priority”. (ref. ^83^, p.755).

**Fig. 4:**
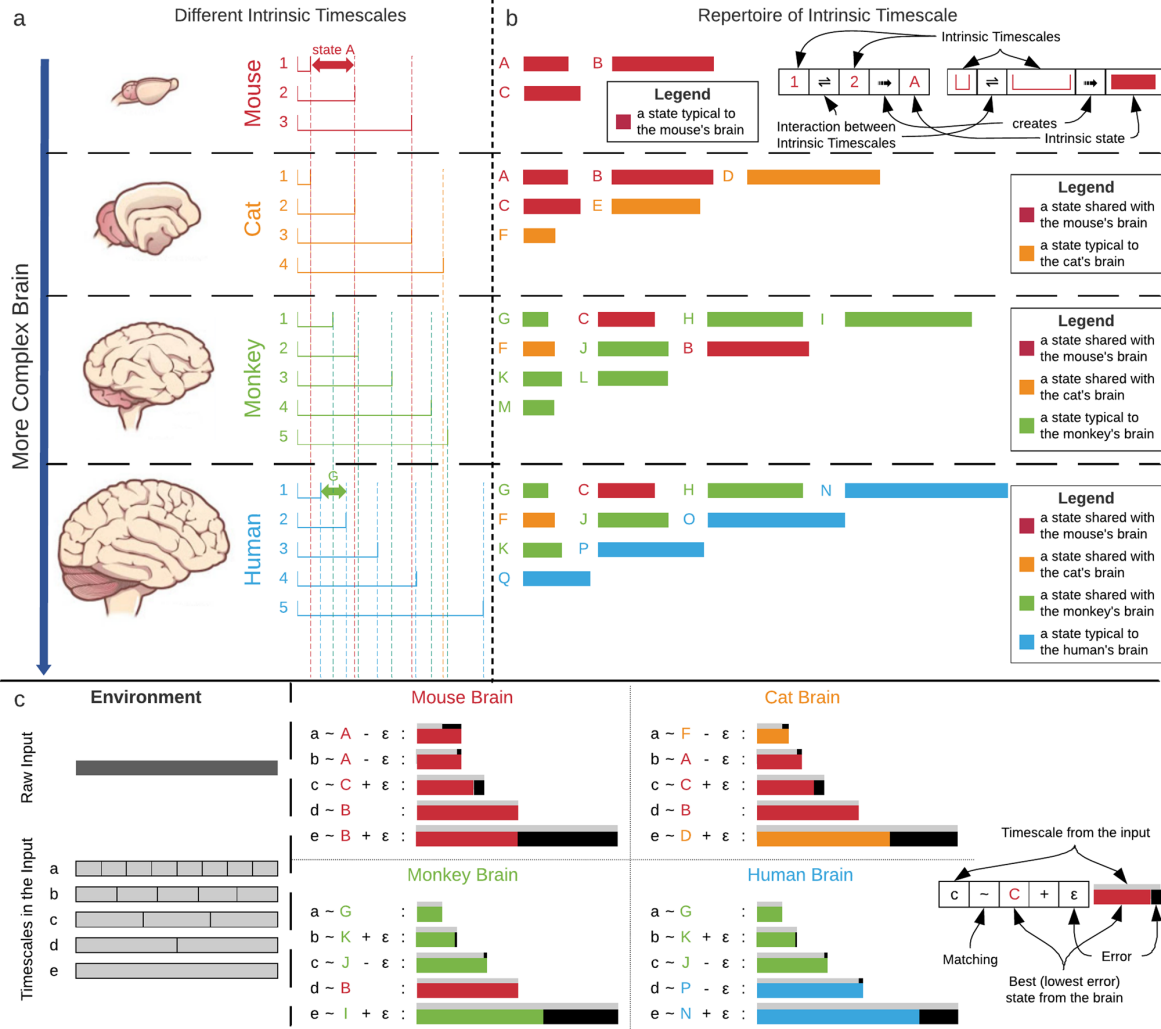
Evolutionary cross-species stochastic matching of the input. All parts are for illustration purpose only and the sequence of brains is not intended to represent any evolutionary hierarchy. **a** Intrinsic timescales in the brains’ of four sample species. On a general scale, a more complex brain has higher number of intrinsic timescales (e.g., human vs. mouse). Also, different brains may have timescales with similar or different lengths. **b** The interaction between different intrinsic timescales may create species’ repertoire of timescales. Each state in the repertoire is the result of the interaction between a pair of timescales. For example, state A is the result of the interaction between timescales 1 and 2. Here, for the sake of simplicity the interaction is defined as the difference between the lengths of two timescales. Although, the timescales themselves are unique to each species’ brain, the interactions (states) can be shared between different species, e.g., state C is shared between all four sample species. So, the repertoire of states in each species’ consists of some states that are typical to that species and some states that are shared with other species. **c** The interaction between the environment and the brain happens through the matching of timescales. On the left, we have the environment and a sample input which contains several timescales with different lengths (a, b, c, d, e). On the right, the matching between the input and each species’ brain is illustrated. Each timescale in the input is matched to the best state from the repertoire of timescales. The best state is the state that yields the least error. The brain clip arts are credited to ref. ^115^.

Is the human brain’s INT an evolutionarily preserved manifestation of our ancestors’ timescales, including their key role in processing external inputs from the environment? The findings by both Shinomoto and colleagues^81,82^ and Buzsáki and colleagues^83^ suggest exactly that. One would consequently expect that human behavior, if based upon its evolutionarily preserved INT, should resemble the behavior of non-human species. For instance, Zhang and Ghazanfar^85^ propose that the timescales of human infant vocal production can be seen in line with the multiple INTs of vocal production in marmoset monkeys, songbirds, and other vertebrates. Together, these findings suggest that INT is, in part, preserved evolutionarily across different species which may be manifested in somewhat similar forms of behavior (Fig. 4a).

### Input encoding: matching the environment’s stochastics by the brain’s intrinsic neural timescales

How is it possible that different species share their INT? The reason for such cross-species similarity cannot be found in the brain itself as cross-species temporal similarities occur across different brain sizes^83^. Picking up the suggestion by Shinomoto and colleagues^81,82^, cross-species similarity may rather be related to similarity in function and, as we specify, in the nature of the inputs. Different species share more or less the same environmental context, i.e., ecological niche and affordances^86–88^. They consequently are exposed to the same input that share similar timescales. Specifically, even if the inputs themselves are elaborated in somewhat distinct ways by the species-specific sensory organs, the input stochastics, i.e., the relation between inputs, may nevertheless be processed in similar ways across the different species.

Is the brain’s INT related to the input stochastics in the environment? Stephens and colleagues^15^ demonstrate that regional differences in the brain’s INT, as indexed in their study by the power spectrum, are related to the temporal structure of the external information, with the former aligning to the latter: (I) regions with shorter INT and faster dynamics, i.e., early auditory cortex, are activated during shorter stimulus segments (e.g., single phonemes or words) (see also refs. ^89–91^ for more support in terms of entrainment); (II) regions with intermediate timescales and balanced slow–fast dynamics, i.e., superior temporal gyrus and inferior frontal gyrus, are recruited by intermediate durations in the temporal structure of stimuli (e.g., the structure of sentences); and (III) regions with longer intrinsic timescales and slower dynamics, i.e., precuneus and medial prefrontal cortex, are activated by slowly varying stimulus dynamics (e.g., stimulus narrative, see also refs. ^1,15,26,27,36^).

These findings raise yet another question, though. How can the limited number of INT of the brain’s various regions process and sample a seemingly unlimited and constantly changing number of extrinsic neural timescales of the environment? Given that there is a positive relation of intra-regional INT and inter-regional functional connectivity^13,18,36^ (see above in Part I), we propose direct interaction between the different regions’ INT—such interaction would enlarge the number of possible timescales, i.e., the repertoire of timescales, as we say (Fig. 4b). We can take the structure of DNA as an analogy. This complex structure is created from only four bases of adenine, thymine, guanine and cytosine. If a species has a high number of regions with different INT, their degree of possible interaction through inter-regional functional connectivity is much higher than in a species with only a low number of regions exhibiting distinct INT and/or low inter-regional functional connectivity.

A large repertoire of timescales may extend the organism’s ability to encode and sample the input stochastics of their respective environment in a more fine-grained and temporally differentiated way, that is, according to distinct timescales in the environment. That, in turn, may reduce the error in the brain’s encoding of the input stochastics relative to the latter’s stochastic occurrence in the natural world. Accordingly, we tentatively suppose that species with a higher number and thus large repertoire of INT are prone to lower degrees of error in their input processing—they can better align to their environmental context in a more fine-grained way than species with a low number of intrinsic timescales and/or a small repertoire (Fig. 4c).

## Part III: mechanisms of input processing through intrinsic neural timescales

### Input segregation and integration: temporal precision vs. smoothing

What are the mechanisms by which the INT processes input? The various task state studies conducted by Hasson and colleagues with the formulation of the temporal receptive windows suggest that the INT may structure the inputs into segments of different durations, e.g., short and long segments like single words, sentence, and paragraphs^1,15,26,27,92^. Such temporal structuring may mean that certain inputs are processed together with high degrees of temporal integration, amounting to some form of “temporal smoothing”^92,93^. Other inputs may be processed in a more segregated and, therefore, temporally precise way entailing higher degrees of temporal segregation (see refs. ^93,94^). Together, this amounts to a balance of temporal integration vs. segregation in input processing.

**Fig. 5:**
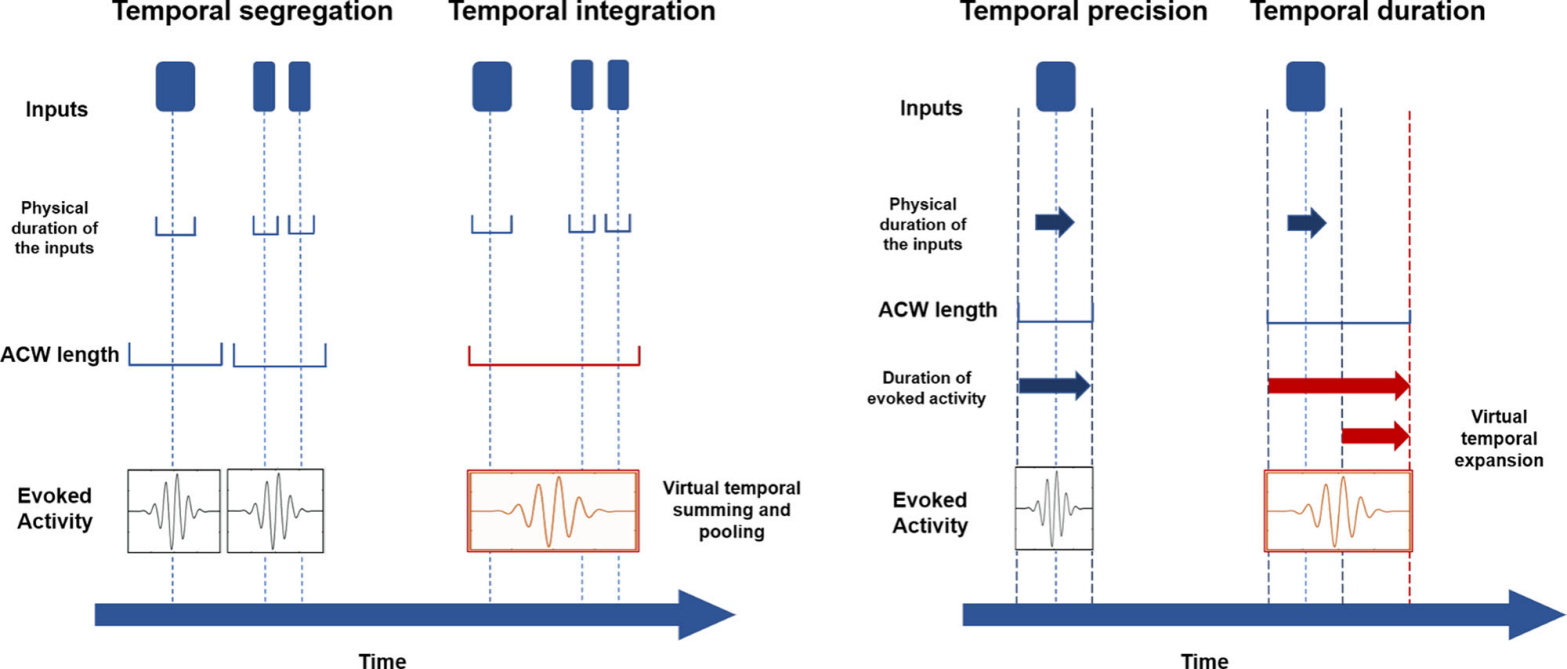
Temporal integration vs. segregation and temporal precision vs. duration in task-evoked activity. The figure highlights two ways how the intrinsic neural timescales can manipulate input processing; this concerns the degree of integration vs. segregation of two (or three) different stimuli (left part) and the degree to which the temporal duration of the stimulus itself can be expanded in neural activity through longer time windows such that the duration of the neuronal activity related to the stimulus, i.e., neuronal duration, is extended beyond the stimulus’ physical duration (right part). From left to right, the figure shows how shorter ACW (especially in unimodal regions) permits to distinguish fast stimuli (high degree of segregation) with a precise temporal encoding consistent with the physical duration of the stimuli (high temporal precision associated with short evoked activity). On the other hand, longer ACW (especially in transmodal regions) permits higher correlation of neural activity across time (high degree of integration), leading to the virtual expansion of the actual stimulus (high temporal duration associated with long evoked activity), i.e., the capacity to encode different stimuli in a way that the evoked activity is longer than the actual physical duration of stimulus.

How can INT modulate their balance of temporal integration vs. segregation during input processing? The ACW measures the degree of correlation of neural activity patterns across different time points. If only a low number of distinct time points correlate with each other, the correlation is low, indexing a short ACW. This means that inputs at more time points beyond those that correlate in ACW are sampled independent of each other—they will be processed with high degrees of temporal segregation but low temporal integration^93^. Moreover, the processing of single inputs may then be more or less restricted to their actual durations as, due to low correlation with a low ACW, they are not expanded (in a virtual way) beyond their actual physical durations, i.e., temporal smoothing or expansion^95^. Accordingly, short INT predisposes that inputs are processed with high temporal precision in both their specific time points and actual durations with low degrees of “temporal smoothing”. Such a pattern of input processing is strongly supported by the short duration of the intrinsic timescales in unimodal regions like the sensory cortex that display a short ACW in rest and a short temporal receptive window during task states^1–3,5,12,15^ (Fig. 5).

The reverse pattern of input processing appears to hold in transmodal regions; their rest ACW and task temporal receptive windows are much longer than those in unimodal regions^1–3,5,12,15^. A longer ACW indicates a higher correlation of neural activity across temporally more distant time points. Inputs at different time points are then not sampled independently of each other, but somewhat linked together across time resulting in “temporal summing and pooling” (see ref. ^93^ who speak of temporal pooling and summing) and ultimately high degrees of temporal integration^93^. Moreover, the duration of the single inputs’ neural processing may virtually, i.e., neuronally, expand beyond their actual physical duration: even if the stimulus is already physically absent (but still present in the neural activity), distant time points’ neural activities may still correlate highly with the preceding time points of the actual stimulus—this amounts to high temporal smoothing/expansion and low temporal precision^95^. For instance, higher-order transmodal regions like the prefrontal cortex support temporal integration and expansion of sensory^51,96–99^, motor, and cognitive information^1,4,6,100^. This is compatible with the idea that transmodal regions such as the prefrontal cortex is involved in higher-order cognition like memory, imagination, abstraction, self, and consciousness.

### Input sampling I: immediate and short vs. delayed and long responses in unimodal and transmodal regions

So far, we have demonstrated how the INT processes the input in particular timescales by modulating them through temporal integration and segregation. However, the brain is confronted with a variety of different inputs in various or multiple scales, with the number of timescales in the environment far exceeding the available timescales of the brain^83,101,102^. How can the brain bridge the gap between its own restricted timescales and the more expanded ones of its environmental context? Ideally, the brain encodes all inputs from the larger-scale environment within its own smaller-scale neural activity without losing any information, i.e., minimal error.

The empirical data show hierarchical organization of the timescales within the brain according to a fast–slow gradient from uni- to transmodal regions. The inputs may be sampled in a more or less analogous way when transitioning from the faster unimodal to the slower transmodal regions; this implies what in mathematics is described as down-sampling from faster input stochastics to slower ones^103^. We suggest that the INT acts as input samplers, that is, down-sampling across the hierarchy of unimodal and transmodal regions. Here, we first perform numerical simulations to provide some support for the differential response of unimodal and transmodal regions during input processing (this section). This will be complemented in a second step (next section) by illustrating the mathematical principles of the fast–slow gradient of down-sampling again showing some simulation data.

Under our fast–slow gradient assumption, sensory networks would be the first to carry out this down-sampling process. Unimodal and sensory networks show shorter intrinsic timescales compared to transmodal ones^15,22^. This implies that the first sampling would be done at a higher frequency and, progressively, said sampling would be on more widely spaced timescales, i.e., down-sampling. In that case, one would expect a faster and more transient, i.e., fast-frequency response in unimodal regions as related to their shorter INT. Transmodal regions, in contrast, should show a slower, delayed and longer-lasting response.

**Fig. 6:**
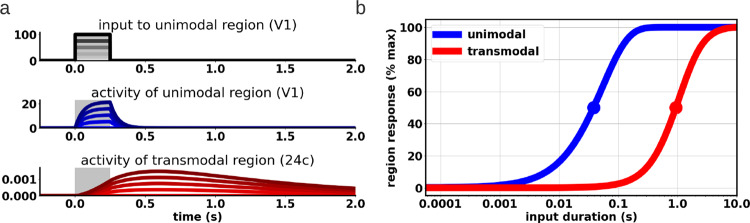
Distinct neural timescales from input perturbations. **a** Activity of primary visual cortex (V1) and anterior cingulate cortex (24c) in response to 250 ms of pulse input of varying strengths to area V1. The unimodal region V1 exhibits fast, short-lasting responses, whereas the transmodal region 24c exhibits slower, long-lasting responses. **b** Input duration is differentially regulated by unimodal and transmodal regions. The unimodal region V1 shows rapid response saturation to brief input durations, reflecting fast integration of sensory-relevant stimuli. The response of the transmodal region 24c saturates over longer timescales, reflecting slow and delayed temporal integration of inputs.

In a first step, we probed this in a computational network model^5^ (details are provided in the Supplementary material), applying inputs of short duration to lower-order regions, i.e., visual cortex V1, and tracking the response in both lower- and higher-order regions, i.e., anterior cingulate cortex 24c. Indeed, we observe immediate and short-lasting responses in V1, whereas 24c displays more delayed, i.e., sluggish and longer-lasting responses (Fig. 6a). This is compatible with a fast–slow gradient of consecutive input down-sampling throughout the lower-higher hierarchical processing stages.

In a second step, we varied the duration of the input to V1. Inputs of shorter duration should yield a faster response saturation in lower-order regions like V1 while inputs of longer duration would be required to yield the same effect in the higher-order regions like 24c. This again was confirmed in our simulation data (Fig. 6b), providing indirect support for input down-sampling along the lines of a fast–slow gradient. In sum, there seems to be a close link of fast–slow input sampling during the encoding of the input stochastics, with the latter being sampled in a seemingly temporal way by the brain’s INT.

Together, these simulation results are compatible with a recent study by Wengler and colleagues^25^. They investigate fMRI-based ACW in subsequent regions of three sensory-based input streams, sensorimotor, visual, and auditory. In their data, the ACW shows a short to long gradient from primary over secondary sensory to higher-order sensory regions (like frontal eye field and dorsolateral prefrontal cortex). This holds for all three sensorimotor, visual, and auditory systems. Albeit indirectly, this supports our view of the fast–slow gradient mediating the continuous down-sampling of the input when transitioning from lower-order unimodal to higher-order transmodal regions.

### Input sampling II: unimodal–transmodal hierarchy mediates fast–slow gradient of down-sampling

**Fig. 7:**
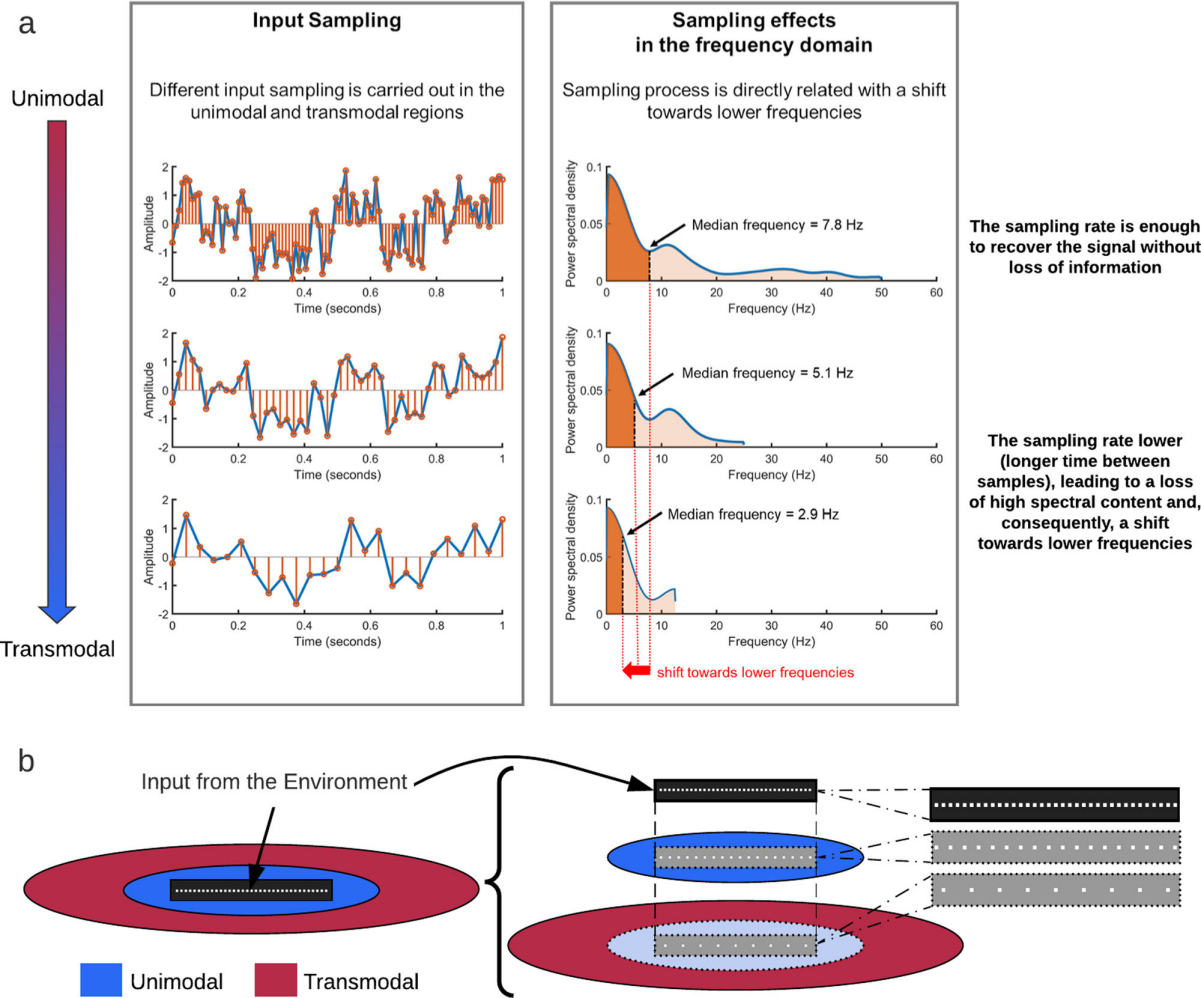
Input sampling in the uni- and transmodal units. **a** The sub-sampling of a signal and shift in the frequency. The more the input signal is sampled, the more shift toward slower frequencies (indexed by median frequency). The first of the three plots shows the original signal with sampling frequency of 100 Hz in time (left) and frequency (right) domains. The second row shows the same signal after sub-sampled, that is, the new sampling frequency is 50 Hz. The spectral component of this signal is shifted toward slower frequency compared to the previous one. Third row shows the same signal, but after a new sub-sampling at 25 Hz. **b** The same concept as (**a**) in the brain’s uni- and transmodal regions. The input is processed by both unimodal and transmodal regions, each time passing from a sampling machine, thus shifting toward slower frequencies (slow mode).

Mathematically speaking, the fast–slow gradient with continuous down-sampling entails a shift from faster to slower frequencies in the input stochastics, that is, a decrease in its maximum interpretable frequency. The more input processing advances towards the transmodal end of the fast–slow gradient, the more its fast frequency-based temporal precision decreases. This means that different inputs may no longer be distinguishable from each other, something which, in signal processing, is described as aliasing^103^ (Fig. 7a right). Accordingly, the stronger the down-sampling going along with lower sampling rate of the input, the slower the maximum frequency that can be reconstructed from a discrete signal, i.e., a signal after a sampling process^103,104^.

On the neuronal side, down-sampling along the fast–slow gradient of the input stochastics results in a shift of the spectral content towards slower frequencies—this can be easily indexed by the median frequency. In fact, for signals where most of the spectral content is found in slow frequencies, as in brain’s fMRI and EEG / MEG signals, this is exactly what one can observe. They have a 1⁄*f* distribution with stronger power in slow frequencies and less power in faster frequencies.

Analogously, the down-sampling process carried out throughout the unimodal–transmodal hierarchy of the INT necessarily leads to a removal of the faster frequencies to achieve good resolution of the inputs’ slow frequencies^103,104^. Throughout the course of the unimodal to transmodal hierarchical processing, one loses the inputs’ information in the faster frequencies but preserves its disproportionally strong slow frequency information (see Fig. 7a for a simulation of this model using pink noise). A reduction in the sampling frequency causes the information to be maintained in slower frequencies with their higher spectral power at the cost of losing the detailed information provided by the faster frequencies. In other words, the original inputs may be shifted towards the slower frequencies in the transmodal regions. Down-sampling along a fast–slow gradient may thus be optimal for preserving the maximal amount of input information along its whole temporal range including both fast and slow frequency input components.

The assumption of such fast–slow down-sampling along the unimodal–transmodal hierarchy is compatible with the empirical data, that is, the relation of ACW with spectral content, i.e., the power spectrum. The longer ACW in transmodal regions are related to stronger power in infraslow frequencies as compared with faster ones. Shorter ACWs in unimodal regions are more dominated by their (relative) shift towards faster frequencies, meaning that their median frequency is lower^12,15,44,65,105,106^ (Fig. 7b). This raises the question for the role or function of slower frequencies like delta (1–4 Hz), slow cortical potentials in the 0.1–1 Hz range, and the infraslow frequencies (0.001–0.1 Hz) in input processing. Unlike faster frequencies like gamma (>30 Hz), beta (14–30 Hz), alpha, and theta^101,102^, the role or function of these slower frequencies is currently unclear (see also refs. ^106–108^).

We know that INT is closely linked to slow frequencies and their long cycle durations (see also refs. ^12,21^). Longer cycle durations means that more inputs can be temporally integrated within one cycle and subsequently be processed together. The longer INT, especially in the transmodal regions, thus samples^12,21^ the inputs in favor of a slow mode—the originally faster inputs are filtered and processed in a slow frequency way, i.e., sub-sampling (see refs. ^12,13,15,93,108–110^; see also ref. ^107^). On a more cognitive level, this means that internally oriented cognition like mind-wandering^111^ or mental time travel^112^, as mediated by transmodal regions, may be characterized by predominant slow modes (see also ref. ^113^).

One may now raise yet another question. We assumed that the INT is key in input encoding, that is, encoding the stochastics of the input. One would now assume that such input encoding should be closely related to the input sampling in terms of fast–slow down-sampling. Do input encoding and encoding sampling converge? This is indeed supported by a recent combined human EEG and modeling study. SanCristobal and colleagues^114^ show that the neural processing of the input stochastics of a looming sound (3 s) is directly related to the resting state’s ACW: the longer the resting state’s ACW, the better the task-related power changes in delta, alpha, and beta could track the physical dynamics of the looming sound. This supports the assumption that the resting state’s INT display the capacity for input sampling with the consequent bias towards the slow frequency mode.

Next, SanCristobal et al.^114^ complement these data by a computational model probing for the slower mode (via Ornstein-Uhlenbeck process): a longer ACW exerts a lower sampling frequency with a shift towards slower frequencies in input sampling. This in turn makes it impossible to obtain information from faster frequencies. Together, these findings support the notion that INT mediates input sampling by tilting or biasing it towards the slow frequency mode.

Even more important, these findings suggest that the seemingly stochastic nature of input encoding converges with the fast–slow gradient of down-sampling. Is the fast–slow gradient of down-sampling, with the emphasis on slow-frequency encoding, the best way of bridging the timescale differences of the brain and environment? The timescale of the environment exceeds far beyond that of the brain, especially in the slow frequency ranges (as, for instance the brain cannot process the ultra-slow frequency ranges of seismic earth waves). The fast–slow gradient of down-sampling may then best be suitable for overcoming the timescale differences between the environmental context (where the input is coming from) and the brain in order to encode best the former’s slow frequency modes.

## Conclusion

How can the brain process temporally complex inputs such as music and language and, even better, integrate them into one meaningful whole as, for instance, a melody or a sentence? We propose that the intrinsic neural timescales (INT) take on a key role or function for the brain’s input processing. This is supported by the major role of INT in both resting and task states, including carry-over from rest to task. Following these findings, we propose that a key function of the INT consists in the dynamic shaping and structuring of input processing, including its different facets like cross-species input sharing and encoding of input stochastics. This concerns input sharing across species as well as input encoding through matching the stochastics of both environment and brain. While that may be mediated by two key mechanisms, (I) input integration vs. segregation on temporal grounds as well as (II) fast–slow down-sampling along the unimodal–transmodal hierarchy of the INT. Taken together, their key role in input processing through distinct mechanisms renders INT highly relevant for current views of the brain’s function, including its role in mental features and psychiatric disorders (Box 2), as well as for designing artificial intelligence (Box 3).

## Supplementary information

Supplementary material.

## Data Availability

The scripts that support the simulation and computational models of this study are freely available at www.georgnorthoff.com/codes.

## References

[1] Hasson U, Chen J, Honey CJ (2015). Hierarchical process memory: memory as an integral component of information processing. Trends Cogn. Sci..

[2] Gollo LL, Roberts JA, Cocchi L (2017). Mapping how local perturbations influence systems-level brain dynamics. NeuroImage.

[3] Gollo LL (2015). Dwelling quietly in the rich club: brain network determinants of slow cortical fluctuations. Philos. Trans. R. Soc. B.

[4] Murray JD (2014). A hierarchy of intrinsic timescales across primate cortex. Nat. Neurosci..

[5] Chaudhuri R, Knoblauch K, Gariel MA, Kennedy H, Wang XJ (2015). A large-scale circuit mechanism for hierarchical dynamical processing in the primate cortex. Neuron.

[6] Farzan F (2017). Brain temporal complexity in explaining the therapeutic and cognitive effects of seizure therapy. Brain.

[7] Deco, G., Cruzat, J. & Kringelbach, M. L. Brain songs framework used for discovering the relevant timescale of the human brain. *Nat. Commun.* **10**, 583 (2019). 10.1038/s41467-018-08186-7 PMC6361902 30718478

[8] Liégeois, R. et al. Resting brain dynamics at different timescales capture distinct aspects of human behavior. *Nat. Commun.* **10**, 2317 (2019). 10.1038/s41467-019-10317-7 PMC6534566 31127095

[9] Wasmuht DF, Spaak E, Buschman TJ, Miller EK, Stokes MG (2018). Intrinsic neuronal dynamics predict distinct functional roles during working memory. Nat. Commun..

[10] Yeshurun, Y., Nguyen, M. & Hasson, U. The default mode network: where the idiosyncratic self meets the shared social world. *Nat. Rev. Neurosci.* **22**,181–192 (2021). 10.1038/s41583-020-00420-w PMC7959111 33483717

[11] Chien, H. Y. S. & Honey, C. J. Constructing and forgetting temporal context in the human cerebral cortex. *Neuron* **106**, 675–686.e11 (2020). 10.1016/j.neuron.2020.02.013 PMC7244383 32164874

[12] Honey CJ (2012). Slow cortical dynamics and the accumulation of information over long timescales. Neuron.

[13] Raut RV (2020). Organization of propagated intrinsic brain activity in individual humans. Cereb. Cortex.

[14] Watanabe, T., Rees, G. & Masuda, N. Atypical intrinsic neural timescale in autism. *eLife* **8**, e42256 (2019). 10.7554/eLife.42256 PMC6363380 30717827

[15] Stephens, G. J., Honey, C. J. & Hasson, U. A place for time: the spatiotemporal structure of neural dynamics during natural audition. *J. Neurophysiol.* **110**, 2019–2026 (2013). 10.1152/jn.00268.2013 PMC3841928 23926041

[16] Chaudhuri, R., Bernacchia, A. & Wang, X.-J. A diversity of localized timescales in network activity. *eLife* **3**, e01239 (2014). 10.7554/eLife.01239 PMC3895880 24448407

[17] Kiebel SJ, Daunizeau J, Friston KJ (2008). A hierarchy of time-scales and the brain. PLoS Comput. Biol..

[18] Ito, T., Hearne, L. J. & Cole, M. W. A cortical hierarchy of localized and distributed processes revealed via dissociation of task activations, connectivity changes, and intrinsic timescales. *NeuroImage* **221**, 117141 (2020). 10.1016/j.neuroimage.2020.117141 PMC7779074 32663642

[19] Burt, J. B. et al. Hierarchy of transcriptomic specialization across human cortex captured by structural neuroimaging topography. *Nat. Neurosci.* **21**, 1251–1259 (2018). 10.1038/s41593-018-0195-0 PMC6119093 30082915

[20] Runyan CA, Piasini E, Panzeri S, Harvey CD (2017). Distinct timescales of population coding across cortex.. Nature.

[21] Zilio F (2021). Are intrinsic neural timescales related to sensory processing? Evidence from abnormal behavioral states. NeuroImage.

[22] Golesorkhi M, Gomez-Pilar J, Tumati S, Fraser M, Northoff G (2021). Temporal hierarchy of intrinsic neural timescales converges with spatial core-periphery organization. Commun. Biol..

[23] Shafiei, G. et al. Topographic gradients of intrinsic dynamics across neocortex. *eLife* **9**, e62116 (2020). 10.7554/eLife.62116 PMC7771969 33331819

[24] Gao R, van den Brink RL, Pfeffer T, Voytek B (2020). Neuronal timescales are functionally dynamic and shaped by cortical microarchitecture. eLife.

[25] Wengler, K., Goldberg, A. T., Chahine, G. & Horga, G. Distinct hierarchical alterations of intrinsic neural timescales account for different manifestations of psychosis. *eLife* **9**, e56151 (2020). 10.7554/eLife.56151 PMC7591251 33107431

[26] Chen, J., Hasson, U. & Honey, C. J. Processing timescales as an organizing principle for primate cortex. *Neuron* **88**, 244–246 (2015). 10.1016/j.neuron.2015.10.010 26494274

[27] Chen J (2017). Shared memories reveal shared structure in neural activity across individuals. Nat. Neurosci..

[28] Cavanagh, S. E., Wallis, J. D., Kennerley, S. W. & Hunt, L. T. Autocorrelation structure at rest predicts value correlates of single neurons during reward-guided choice. *eLife* **5**, e18937 (2016). 10.7554/eLife.18937 PMC5052031 27705742

[29] Demirtaş M (2019). Hierarchical heterogeneity across human cortex shapes large-scale neural dynamics. Neuron.

[30] Jansen BH, Rit VG (1995). Electroencephalogram and visual evoked potential generation in a mathematical model of coupled cortical columns. Biol. Cybern..

[31] David O, Friston KJ (2003). A neural mass model for MEG/EEG. NeuroImage.

[32] Kaplan HS, Salazar Thula O, Khoss N, Zimmer M (2020). Nested neuronal dynamics orchestrate a behavioral hierarchy across timescales. Neuron.

[33] Feng NY, Fergus DJ, Bass AH (2015). Neural transcriptome reveals molecular mechanisms for temporal control of vocalization across multiple timescales. BMC Genomics.

[34] Beiran M, Ostojic S (2019). Contrasting the effects of adaptation and synaptic filtering on the timescales of dynamics in recurrent networks. PLoS Comput. Biol..

[35] Cocchi L, Gollo LL, Zalesky A, Breakspear M (2017). Criticality in the brain: a synthesis of neurobiology, models and cognition. Prog. Neurobiol..

[36] Cavanagh, S. E., Hunt, L. T. & Kennerley, S. W. A diversity of intrinsic timescales underlie neural computations. *Front. Neural Circuits* **14**, 615626 (2020). 10.3389/fncir.2020.615626 PMC7779632 33408616

[37] Spitmaan M, Seo H, Lee D, Soltani A (2020). Multiple timescales of neural dynamics and integration of task-relevant signals across cortex. Proc. Natl Acad. Sci. USA.

[38] Soltani A, Murray JD, Seo H, Lee D (2021). Timescales of cognition in the brain. Curr. Opin. Behav. Sci..

[39] Sadaghiani S, Wirsich J (2020). Intrinsic connectome organization across temporal scales: new insights from cross-modal approaches. Netw. Neurosci..

[40] Kaneoke Y (2012). Variance and autocorrelation of the spontaneous slow brain activity. PLoS ONE.

[41] Ogawa T, Komatsu H (2010). Differential temporal storage capacity in the baseline activity of neurons in macaque frontal eye field and area V4. J. Neurophysiol..

[42] Fallon J (2020). Timescales of spontaneous fMRI fluctuations relate to structural connectivity in the brain. Netw. Neurosci..

[43] Ito T, Hearne L, Mill R, Cocuzza C, Cole MW (2020). Discovering the computational relevance of brain network organization. Trends Cogn. Sci..

[44] Baria, A. T. et al. Linking human brain local activity fluctuations to structural and functional network architectures. *NeuroImage* **73**, 144–155 (2013). 10.1016/j.neuroimage.2013.01.072 PMC3632346 23396160

[45] Menceloglu M, Grabowecky M, Suzuki S (2020). EEG state-trajectory instability and speed reveal global rules of intrinsic spatiotemporal neural dynamics. PLoS ONE.

[46] Kucyi A, Davis KD (2015). The dynamic pain connectome. Trends Neurosci..

[47] Honari H, Choe AS, Pekar JJ, Lindquist MA (2019). Investigating the impact of autocorrelation on time-varying connectivity. NeuroImage.

[48] Salvador R (2008). A simple view of the brain through a frequency-specific functional connectivity measure. NeuroImage.

[49] Regev M (2019). Propagation of information along the cortical hierarchy as a function of attention while reading and listening to stories. Cereb. Cortex.

[50] Nguyen M, Vanderwal T, Hasson U (2019). Shared understanding of narratives is correlated with shared neural responses. NeuroImage.

[51] Yeshurun Y, Nguyen M, Hasson U (2017). Amplification of local changes along the timescale processing hierarchy. Proc. Natl Acad. Sci. USA.

[52] Cole MW (2013). Multi-task connectivity reveals flexible hubs for adaptive task control. Nat. Neurosci..

[53] Cole MW, Yarkoni T, Repovs G, Anticevic A, Braver TS (2012). Global connectivity of prefrontal cortex predicts cognitive control and intelligence. J. Neurosci..

[54] Tavor I (2016). Task-free MRI predicts individual differences in brain activity during task performance. Science.

[55] Ito T (2017). Cognitive task information is transferred between brain regions via resting-state network topology. Nat. Commun..

[56] Northoff G, Qin P, Nakao T (2010). Rest-stimulus interaction in the brain: a review. Trends Neurosci..

[57] Huang Z (2017). Is there a nonadditive interaction between spontaneous and evoked activity? Phase-dependence and its relation to the temporal structure of scale-free brain activity. Cereb. Cortex.

[58] Northoff G, Duncan NW, Hayes DJ (2010). The brain and its resting state activity—experimental and methodological implications. Prog. Neurobiol..

[59] Wainio-Theberge, S., Wolff, A. & Northoff, G. Bridging the gap – spontaneous fluctuations shape stimulus-evoked spectral power. *bioRxiv*10.1101/2020.06.23.166058 (2020).

[60] Sarracino A, Arviv O, Shriki O, de Arcangelis L (2020). Predicting brain evoked response to external stimuli from temporal correlations of spontaneous activity. Phys. Rev. Res..

[61] Gollo, L. L. Exploring atypical timescales in the brain. *eLife* **8**, e45089 (2019). 10.7554/eLife.45089 PMC6363382 30717825

[62] Northoff, G. & Gomez-Pilar, J. Overcoming rest–task divide—abnormal temporospatial dynamics and its cognition in schizophrenia. *Schizophr. Bull.* **47**, 751–765 (2021). 10.1093/schbul/sbaa178 PMC8661394 33305324

[63] Cirillo R, Fascianelli V, Ferrucci L, Genovesio A (2018). Neural intrinsic timescales in the macaque dorsal premotor cortex predict the strength of spatial response coding. iScience.

[64] Huang Z (2018). Disrupted neural variability during propofol-induced sedation and unconsciousness. Hum. Brain Mapp..

[65] Huang Z, Liu X, Mashour GA, Hudetz AG (2018). Timescales of intrinsic BOLD signal dynamics and functional connectivity in pharmacologic and neuropathologic states of unconsciousness. J. Neurosci..

[66] Wolff A (2019). The temporal signature of self: temporal measures of resting-state EEG predict self-consciousness. Hum. Brain Mapp..

[67] Northoff G (2017). Personal identity and cortical midline structure (CMS): do temporal features of cms neural activity transform into “Self-Continuity”?. Psychol. Inq..

[68] Kolvoort, I. R., Wainio-Theberge, S., Wolff, A. & Northoff, G. Temporal integration as “common currency” of brain and self-scale-free activity in resting-state EEG correlates with temporal delay effects on self-relatedness. *Human Brain Mapp.* **41**, 4355–4374 (2020). 10.1002/hbm.25129 PMC7502844 32697351

[69] Sugimura, K. et al. Association between long-range temporal correlations in intrinsic EEG activity and subjective sense of identity. *Sci. Rep.* **11**, 422 (2021). 10.1038/s41598-020-79444-2 PMC7801398 33431948

[70] Ventriglia F (2014). Random dispersion in excitatory synapse response. Cogn. Neurodyn..

[71] Déli E, Tozzi A, Peters JF (2017). Relationships between short and fast brain timescales. Cogn. Neurodyn..

[72] Sellers KK, Bennett DV, Hutt A, Williams JH, Fröhlich F (2015). Awake vs. anesthetized: layer-specific sensory processing in visual cortex and functional connectivity between cortical areas. J. Neurophysiol..

[73] Schiff ND, Nauvel T, Victor JD (2014). Large-scale brain dynamics in disorders of consciousness. Curr. Opin. Neurobiol..

[74] Pistoia F (2015). Contribution of interoceptive information to emotional processing: evidence from individuals with spinal cord injury. J. Neurotrauma.

[75] Fischer KW, Goswami U, Geake J (2010). The future of educational neuroscience. Mind, Brain, Educ..

[76] Andrillon T, Kouider S (2020). The vigilant sleeper: neural mechanisms of sensory (de)coupling during sleep. Curr. Opin. Physiol..

[77] Northoff, G. *Unlocking the Brain* (Oxford, 2014) 10.1093/acprof:oso/9780199826995.001.0001.

[78] Northoff, G. & Lamme, V. Neural signs and mechanisms of consciousness: is there a potential convergence of theories of consciousness in sight? *Neurosci. Biobehav. Rev.* **118**, 568–587 (2020). 10.1016/j.neubiorev.2020.07.019 32783969

[79] Northoff G, Heiss W-D (2015). Why is the distinction between neural predispositions, prerequisites, and correlates of the level of consciousness clinically relevant?. Stroke.

[80] Northoff G (2013). Gene, brains, and environment—genetic neuroimaging of depression. Curr. Opin. Neurobiol..

[81] Shinomoto S (2009). Relating neuronal firing patterns to functional differentiation of cerebral cortex. PLoS Comput. Biol..

[82] Mochizuki Y (2016). Similarity in neuronal firing regimes across mammalian species. J. Neurosci..

[83] Buzsáki G, Logothetis N, Singer W (2013). Scaling brain size, keeping timing: evolutionary preservation of brain rhythms. Neuron.

[84] Fulcher BD, Murray JD, Zerbi V, Wang X-J (2019). Multimodal gradients across mouse cortex. Proc. Natl Acad. Sci. USA.

[85] Zhang YS, Ghazanfar AA (2020). A hierarchy of autonomous systems for vocal production. Trends Neurosci..

[86] Bruineberg J, Chemero A, Rietveld E (2019). General ecological information supports engagement with affordances for ‘higher’ cognition. Synthese.

[87] Bruineberg, J. & Rietveld, E. Self-organization, free energy minimization, and optimal grip on a field of affordances. *Front. Hum. Neurosci.* **8**, 599 (2014). 10.3389/fnhum.2014.00599 PMC4130179 25161615

[88] Bruineberg, J., Rietveld, E., Parr, T., van Maanen, L. & Friston, K. J. Free-energy minimization in joint agent-environment systems: a niche construction perspective. *J.Theor. Biol.* **455**, 161–178 (2018). 10.1016/j.jtbi.2018.07.002 PMC6117456 30012517

[89] Henao D, Navarrete M, Valderrama M, le Van Quyen M (2020). Entrainment and synchronization of brain oscillations to auditory stimulations. Neurosci. Res..

[90] Lakatos P, Gross J, Thut G (2019). A new unifying account of the roles of neuronal entrainment. Curr. Biol..

[91] Teng X, Tian X, Rowland J, Poeppel D (2017). Concurrent temporal channels for auditory processing: oscillatory neural entrainment reveals segregation of function at different scales. PLoS Biol..

[92] Müsch K, Himberger K, Tan KM, Valiante TA, Honey CJ (2020). Transformation of speech sequences in human sensorimotor circuits. Proc. Natl Acad. Sci. USA.

[93] Himberger KD, Chien HY, Honey CJ (2018). Principles of temporal processing across the cortical hierarchy. Neuroscience.

[94] Zilio, F. et al. Are intrinsic neural timescales related to sensory processing? Evidence from abnormal behavioral states. *NeuroImage* **226**, 117579, 10.1016/j.neuroimage.2020.117579Get (2021). 10.1016/j.neuroimage.2020.117579 33221441

[95] Northoff G, Huang Z (2017). How do the brain’s time and space mediate consciousness and its different dimensions? Temporo-spatial theory of consciousness (TTC). Neurosci. Biobehav. Rev..

[96] Hasson U, Furman O, Clark D, Dudai Y, Davachi L (2008). Enhanced intersubject correlations during movie viewing correlate with successful episodic encoding. Neuron.

[97] Lerner Y, Honey CJ, Silbert LJ, Hasson U (2011). Topographic mapping of a hierarchy of temporal receptive windows using a narrated story. J. Neurosci..

[98] Stephens GJ, Honey CJ, Hasson U (2013). A place for time: the spatiotemporal structure of neural dynamics during natural audition. J. Neurophysiol..

[99] Gauthier B, Eger E, Hesselmann G, Giraud A-L, Kleinschmidt A (2012). Temporal tuning properties along the human ventral visual stream. J. Neurosci..

[100] Bernacchia A, Seo H, Lee D, Wang X-JJ (2011). A reservoir of time constants for memory traces in cortical neurons. Nat. Neurosci..

[101] Buzsáki, G. *Rhythms of the Brain* (Oxford, 2009). 10.1093/acprof:oso/9780195301069.001.0001.

[102] Buzsáki, G. *The Brain from Inside Out* (Oxford, 2019) 10.1093/oso/9780190905385.001.0001.

[103] Oppenheim, A. V. *Signals, Systems and Interference* (Pearson, 2016).

[104] Shannon, C. E. Communication in the presence of noise. *Proc. IRE.* Vol. 37, 10–21 (IEEE, 1949) 10.1109/JRPROC.1949.232969.

[105] Huang Z, Obara N, Davis HH, Pokorny J, Northoff G (2016). The temporal structure of resting-state brain activity in the medial prefrontal cortex predicts self-consciousness. Neuropsychologia.

[106] Golesorkhi, M., Tumati, S., Gomez-Pilar, J., Stamatakis, E. A. & Northoff, G. The interplay between information flux and temporal dynamics in infraslow frequencies. *bioRxiv*10.1101/2020.06.11.106476 (2020).

[107] Northoff G (2017). “Paradox of slow frequencies” – are slow frequencies in upper cortical layers a neural predisposition of the level/state of consciousness (NPC)?. Conscious. Cognition.

[108] Sanchez-Vives MV, Massimini M, Mattia M (2017). Shaping the default activity pattern of the cortical network. Neuron.

[109] He BJ, Raichle ME (2009). The fMRI signal, slow cortical potential and consciousness. Trends Cogn. Sci..

[110] Sanchez-Vives MV, Mattia M (2014). Slow wave activity as the default mode of the cerebral cortex. Arch. Ital. Biol..

[111] Vanhaudenhuyse, A. et al. Two distinct neuronal networks mediate the awareness of environment and of self. *J. Cogn. Neurosci.* **23**, 570–578 (2011). 10.1162/jocn.2010.21488 20515407

[112] Schacter DL (2012). The future of memory: remembering, imagining, and the brain. Neuron.

[113] Deco, G. & Kringelbach, M. L. Hierarchy of information processing in the brain: a novel ‘Intrinsic Ignition’ framework. *Neuron* **94**, 961–968 (2017). 10.1016/j.neuron.2017.03.028 28595052

[114] Sancristóbal, B. et al. Slow Resting State Fluctuations Enhance Neuronal and Behavioral Responses to Looming Sounds. *Brain Topography,* 1–21, 10.1007/s10548-021-00826-4 (2021). 10.1007/s10548-021-00826-4 33768383

[115] OpenStax. The central nervous system. *Biology* (OpenStax CNX, 2020).

[116] Taylor P, Hobbs JN, Burroni J, Siegelmann HT (2015). The global landscape of cognition: hierarchical aggregation as an organizational principle of human cortical networks and functions. Sci. Rep..

[117] Northoff G, Qin P (2011). How can the brain’s resting state activity generate hallucinations? A ‘resting state hypothesis’ of auditory verbal hallucinations. Schizophr. Res..

[118] Northoff G, Duncan NW (2016). How do abnormalities in the brain’s spontaneous activity translate into symptoms in schizophrenia? From an overview of resting state activity findings to a proposed spatiotemporal psychopathology. Prog. Neurobiol..

[119] Christoff K, Irving ZC, Fox KCRR, Spreng RN, Andrews-Hanna JR (2016). Mind-wandering as spontaneous thought: a dynamic framework. Nat. Rev. Neurosci..

[120] Northoff G (2018). The brain’s spontaneous activity and its psychopathological symptoms – “Spatiotemporal binding and integration”. Prog. Neuropsychopharmacol. Biol. Psychiatry.

[121] Northoff G (2006). Self-referential processing in our brain—a meta-analysis of imaging studies on the self. NeuroImage.

[122] Northoff G (2016). Is the self a higher-order or fundamental function of the brain? The “basis model of self-specificity” and its encoding by the brain’s spontaneous activity. Cogn. Neurosci..

[123] Garrett DD, Epp SM, Perry A, Lindenberger U (2018). Local temporal variability reflects functional integration in the human brain. NeuroImage.

[124] Garrett DD, McIntosh AR, Grady CL (2014). Brain signal variability is parametrically modifiable. Cereb. Cortex.

[125] Raichle ME (2001). A default mode of brain function. Proc. Natl Acad. Sci. USA.

[126] Damiani S, Scalabrini A, Gomez-Pilar J, Brondino N, Northoff G (2019). Increased scale-free dynamics in salience network in adult high-functioning autism. NeuroImage: Clin..

[127] Chen J (2016). Accessing real-life episodic information from minutes versus hours earlier modulates hippocampal and high-order cortical dynamics. Cereb. Cortex.

[128] Spronk M (2021). A whole-brain and cross-diagnostic perspective on functional brain network dysfunction. Cereb. Cortex.

[129] Voytek B, Knight RT (2015). Dynamic network communication as a unifying neural basis for cognition, development, aging, and disease. Biol. Psychiatry.

[130] Northoff, G., Sandsten, K. E., Nordgaard, J., Kjaer, T. W. & Parnas, J. The self and its prolonged intrinsic neural timescale in schizophrenia. *Schizophr. Bull.* **47**, 170–179 (2021). 10.1093/schbul/sbaa083 PMC7825007 32614395

[131] Friston K (2013). Active inference and free energy. Behav. Brain Sci..

[132] Fagerholm ED (2020). Conservation laws by virtue of scale symmetries in neural systems. PLoS Comput. Biol..

[133] Yamashita, Y. & Tani, J. Emergence of functional hierarchy in a multiple timescale neural network model: a humanoid robot experiment. *PLoS Comput. Biol.* **4**, e1000220 (2008). 10.1371/journal.pcbi.1000220 PMC2570613 18989398

[134] Paine RW, Tani J (2004). Motor primitive and sequence self-organization in a hierarchical recurrent neural network. Neural Netw..

[135] Choi M, Tani J (2018). Predictive coding for dynamic visual processing: development of functional hierarchy in a multiple spatiotemporal scales RNN model. Neural Comput..

[136] Prescott, T. J. & Camilleri, D. The synthetic psychology of the self. *Cognitive Architectures* 85–104 (Springer, 2019).

[137] Tani J (1996). Model-based learning for mobile robot navigation from the dynamical systems perspective. IEEE Trans. Syst. Man Cybern. B Cybern..

[138] Tani, J. On the interactions between top-down anticipation and bottom-up regression. *Front. Neurorobotics* **1**, 2 (2007). 10.3389/neuro.12.002.2007 PMC2533585 18958273

