# Supplementary material for: The brain and its time: intrinsic neural timescales are key for input processing
Source: Commun Biol. 2021 Aug 16;4:970. doi: 10.1038/s42003-021-02483-6 (PMC8368044; doi:10.1038/s42003-021-02483-6)
Supplement: Supplementary file 1 — Supplementary material. [file 42003_2021_2483_MOESM1_ESM.pdf]

Supplementary Information for

The brain and its time - Intrinsic neural timescales are key for input processing

Details for reproducing figure 6

Figure 6a: Unimodal region V1 was stimulated by an external input ($I_{ext,E}^{i=1}$) pulse for a duration of 250 milliseconds. Input strength was varied from 0 to 100 pA with increments of 25 pA. For each input strength variation, we tracked changes in excitatory population activity in unimodal region V1 ($v_E^{i=1}$) and in transmodal region 24c ($v_E^{i=29}$).

Figure 6b: Unimodal region V1 was stimulation by an external input ($I_{ext,E}^{i=1}$) pulse for a duration of 10 seconds. Input strength was kept constant at 1 pA. At each simulation timestep, we computed the difference in excitatory V1 activity relative to stimulus onset ($v_E^{i=1}(t) - v_E^{i=1}(t_{onset})$). The same procedure was applied over the excitatory population of transmodal region 24c ($v_E^{i=29}(t) - v_E^{i=29}(t_{onset})$). Activity differences in V1 and in 24c were then separately normalized and converted to percentage values – yielding the two dose-response curves.

Details for reproducing the large-scale computational network model

Excitatory and inhibitory population activity
We implemented the large-scale model of hierarchical processing in the primate cortex, considering 29 regions [1]. Each region consists of an excitatory and inhibitory population activity described by:

$$\tau_E \frac{dv_E}{dt} = -v_E + \beta_E [I_E]_+ \tag{1a}$$

$$\tau_I \frac{dv_I}{dt} = -v_I + \beta_I [I_I]_+ \tag{1b}$$

where v_E is the firing rate of the excitatory population, with an intrinsic time constant τ_E and an input current I_E governed by the slope β_E of the f-I curve $[I_E]_+ = \max(I_E, 0)$. The inhibitory population has corresponding parameters v_I, τ_I, I_I, β_I. Parameter values are described below and taken from [1].

Local and long-range inputs to excitatory and inhibitory populations
Each region receives both local (i.e. within area) and long-range (i.e. from other areas) inputs:

$$I_E^i = (1 + \eta h_i)\left(w_{EE} v_E^i + I_{lr,E}^i\right) - w_{EI} v_I^i + I_{ext,E}^i \tag{2a}$$

$$I_I^i = (1 + \eta h_i)\left(w_{IE} v_E^i + I_{lr,I}^i\right) - w_{II} v_I^i + I_{ext,I}^i \tag{2b}$$

where $i \in [1 \dots 29]$ denotes the region index, w_{EE} and w_{EI} are the local excitatory and inhibitory couplings to the excitatory population, respectively. $I_{lr,E}^i$ is the long-range input to the excitatory population and $I_{ext,E}^i$ is the external input to the excitatory population. The inhibitory population has corresponding parameters w_{IE}, w_{II}, $I_{lr,I}^i$, $I_{ext,I}^i$. η is a scaling parameter that controls the effect of the hierarchy.

Hierarchical indices: Both local and long-range inputs to a given region where scaled by the region's position along the hierarchy h_i. Here, h_i was estimated using a sigmoid function restricted between 0 and 1 (Figure S1):

$$h_i = \frac{1}{1 + e^{-x_i}} \tag{3}$$

where x increments linearly, with $\min(x) = -1$, and $\max(x) = 4$.

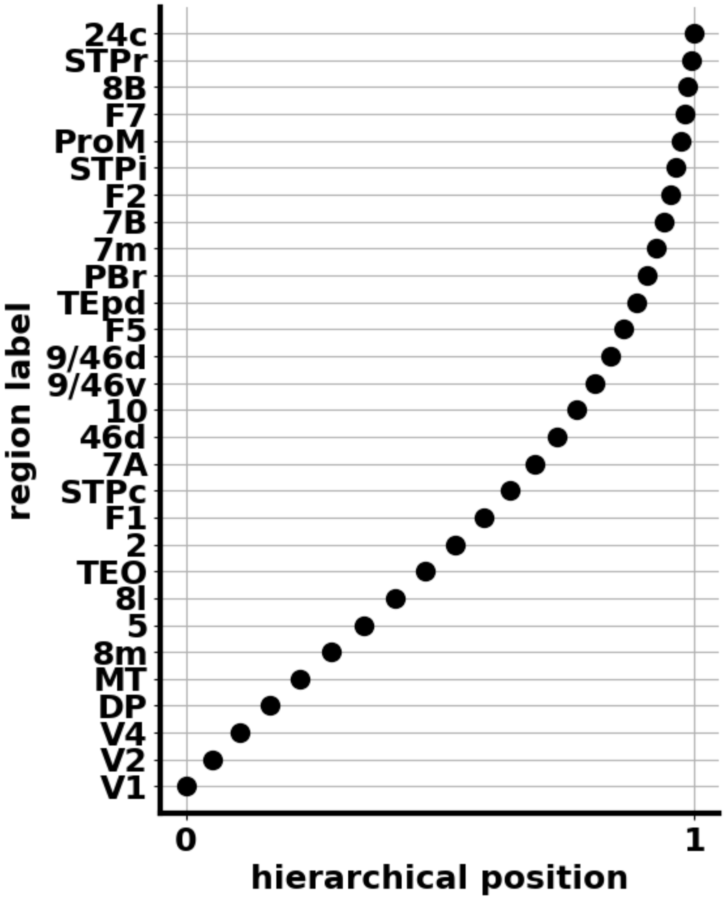

Figure S1. Estimated hierarchy h using a sigmoid function.

Network specificity from structural connectivity

Long-range excitatory inputs to excitatory and inhibitory populations were described by:

$$I_{lr,E}^{i} = \mu_{EE} \sum_{j=1}^{29} J_{ij} v_E^j \tag{4a}$$

$$I_{lr,I}^{i} = \mu_{IE} \sum_{j=1}^{29} J_{ij} v_E^j \tag{4b}$$

where μ_{EE} and μ_{IE} are fixed parameters that control the strength of long-range excitatory input to excitatory and inhibitory populations, respectively. v_E^j is the firing rate of the excitatory population in region j and J_{ij} is the connection strength projecting from region j to region i. As such, J is the structural connectivity matrix, for which its specificity was constructed based on the connectivity data from 29 regions of interest [1] (Figure S2).

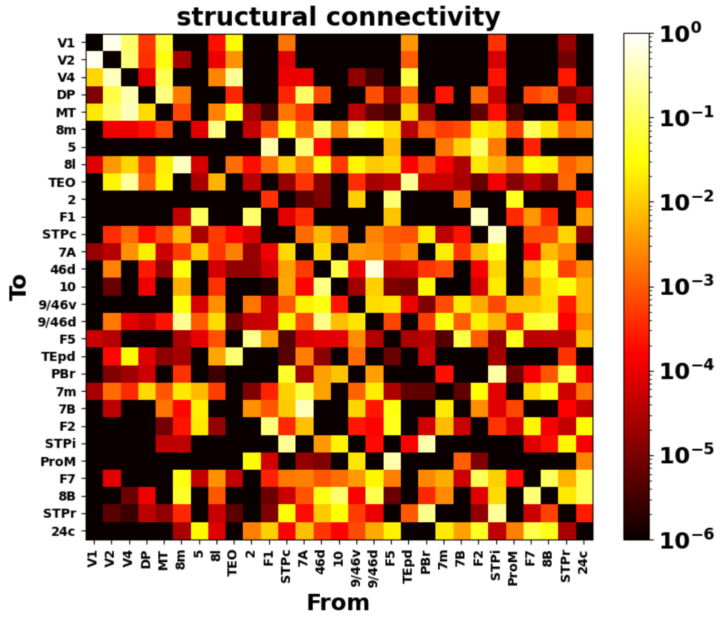

Figure S2. Structural connectivity matrix J from 29 regions.

Model parameters and simulation details

$\tau_E = 20$ ms, $\tau_I = 10$ ms, $\beta_E = 0.066$ Hz/pA, $\beta_I = 0.351$ Hz/pA, $w_{EE} = 24.3$ pA/Hz, $w_{EI} = 19.7$ pA/Hz, $w_{IE} = 12.2$ pA/Hz, $w_{II} = 12.5$ pA/Hz, $\mu_{EE} = 33.7$ pA/Hz, $\mu_{IE} = 25.3$ pA/Hz, $\eta = 0.68$. The differential equations 1a and 1b were solved using the forward Euler method with an integration timestep of 0.1 milliseconds.

References

1. Chaudhuri, R., Knoblauch, K., Gariel, M. A., Kennedy, H. & Wang, X. J. A Large-Scale Circuit Mechanism for Hierarchical Dynamical Processing in the Primate Cortex. *Neuron* (2015) doi:10.1016/j.neuron.2015.09.008.
